# The Effect of Disorder on Endogenous MAS-DNP: Study of Silicate Glasses and Crystals

**DOI:** 10.1021/acs.jpcc.2c08849

**Published:** 2023-02-27

**Authors:** Brijith Thomas, Daniel Jardón-Álvarez, Raanan Carmieli, Johan van Tol, Michal Leskes

**Affiliations:** † Department of Molecular Chemistry & Materials Science, Weizmann Institute of Science, Rehovot 76100, Israel; ‡ Department of Chemical Research Support, Weizmann Institute of Science, Rehovot 76100, Israel; § National High Magnetic Field Laboratory, Florida State University, Tallahassee, Florida 32310, United States

## Abstract

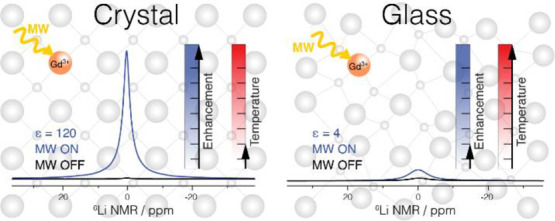

In dynamic nuclear polarization nuclear magnetic resonance (DNP-NMR) experiments, the large Boltzmann polarization of unpaired electrons is transferred to surrounding nuclei, leading to a significant increase in the sensitivity of the NMR signal. In order to obtain large polarization gains in the bulk of inorganic samples, paramagnetic metal ions are introduced as minor dopants acting as polarizing agents. While this approach has been shown to be very efficient in crystalline inorganic oxides, significantly lower enhancements have been reported when applying this approach to oxide glasses. In order to rationalize the origin of the difference in the efficiency of DNP in amorphous and crystalline inorganic matrices, we performed a detailed comparison in terms of their magnetic resonance properties. To diminish differences in the DNP performance arising from distinct nuclear interactions, glass and crystal systems of similar compositions were chosen, Li_2_OCaO·2SiO_2_ and Li_2_CaSiO_4_, respectively. Using Gd(III) as polarizing agent, DNP provided signal enhancements in the range of 100 for the crystalline sample, while only up to around factor 5 in the glass, for both ^6^Li and ^29^Si nuclei. We find that the drop in enhancement in glasses can be attributed to three main factors: shorter nuclear and electron relaxation times as well as the dielectric properties of glass and crystal. The amorphous nature of the glass sample is responsible for a high dielectric loss, leading to efficient microwave absorption and consequently lower effective microwave power and an increase in sample temperature which leads to further reduction of the electron relaxation time. These results help rationalize the observed sensitivity enhancements and provide guidance in identifying materials that could benefit from the DNP approach.

## 1 Introduction

Nuclear magnetic resonance (NMR) spectroscopy provides local structural information at an atomic scale and is, therefore, a unique tool to probe structural properties of materials lacking long-range order. Interactions affecting the nuclear spin properties can be related to structural parameters. Even more, the presence of structural disorder can be mapped from the distribution of the NMR interactions. Due to these properties, solid state NMR plays a fundamental role in the characterization of oxide glasses.^1−4^ The well-known limitation of NMR is its intrinsically low sensitivity, which in silicate glasses is further aggravated by inhomogeneous broadening of the signal, a consequence of local disorder, as well as by the low natural abundance of NMR active isotopes of its main constituents oxygen and silicon. Furthermore, due to the rigidity of the structure, very long longitudinal relaxation times are common in these materials, often impeding the possibility of acquiring multidimensional NMR spectra.

Common strategies for increasing the NMR sensitivity in oxide glasses include isotope enrichment,^5−7^ echo train acquisition,^8,9^ or the addition of small quantities of paramagnetic dopants for paramagnetic relaxation enhancement (PRE).^10−12^ However, isotope labeling can require additional synthesis steps; depending on the sample composition, the enhancement from echo train acquisition might be limited by intrinsically low coherence lifetimes,^13^ and the sensitivity gain from PRE is often not sufficient to avoid long measurement times for high resolution experiments. In recent years we have shown the feasibility of obtaining large NMR signal enhancements for low sensitivity nuclei in the bulk of inorganic oxides by magic angle spinning dynamic nuclear polarization (MAS-DNP).^14−16^ The approach, known as metal ions based (MI)DNP, consists of introducing paramagnetic metal ions as dopants into the sample, which then serve as the source of polarization upon microwave irradiation. Most of the applications, however, were on crystalline oxides. Recently, Paterson et al. extended the use of this methodology to inorganic glasses, obtaining moderate signal enhancements of up to a factor of 4.^17^

By introducing the polarizing agent into the structure of the material of interest itself, the MIDNP approach is conceptually different from the more commonly used exogenous MAS-DNP method.^18^ In the latter, the sample is impregnated with a solution containing nitroxide biradicals, which upon cooling forms a glassy matrix.^19,20^ The DNP enhancements in the exogenous approach are then obtained under microwave irradiation either directly from the unpaired electron spins to the nuclei in the surface of the material or indirectly through the protons in the solvent and subsequent cross-polarization to the nuclei of interest. The formulation of this approach results in surface selective enhancements,^21,22^ and the selectivity will become more pronounced for low sensitivity nuclei in inorganic materials, as spin diffusion, which could transfer polarization into the bulk of the sample, will have limited efficiency. In MIDNP, on the other hand, large enhancements of bulk nuclei can be obtained even in the complete absence of spin diffusion.^23^ Furthermore, enhancements are homogeneous throughout the sample as long as relaxation is governed by the PRE from the polarizing agents themselves.^23^

From these considerations and the fact that glassy matrices are commonly used in exogenous DNP, one could expect the MIDNP approach to yield at least similar enhancements for glassy oxides as for crystalline analogues. A critical parameter determining the efficiency of the MIDNP approach is the distribution of the polarizing agents in the sample. When glasses are formed from rapid quenching of the melt, homogeneous distribution of the dopant in the glass should be ensured. In fact, in the exogenous DNP approach amorphous matrices are desired as they are known to help dispersing the polarizing agents evenly.^24^ Furthermore, glasses present an interesting opportunity for obtaining DNP enhancements via the cross-effect (CE) mechanism.^25^ CE DNP requires a coupled three spin system, including two electrons and one nucleus, where the coupled electrons have a frequency difference equivalent to the nuclear Larmor frequency. When doping crystalline solids, it is most likely that paramagnetic dopants will occupy a unique magnetic equivalent site. This reduces the probability of matching the CE condition within a single crystallite even in the presence of large anisotropies due to alignment of the interaction tensors of equivalent metal ions within each crystal. Thus, MIDNP in crystalline solids most commonly rely on the solid effect (SE) mechanism.^26^ Due to the disordered nature of the glass structure, at high dopant concentrations, the presence of two coupled electrons matching the CE condition becomes feasible and in fact has been suggested based on experimental observations.^17^

In this work, we investigate samples in an amorphous and crystalline state having similar compositions, Li_2_OCaO·2SiO_2_ and Li_2_CaSiO_4_, respectively, doped with Gd(III) at various concentrations. Similar compositions are taken to diminish differences in DNP performance which could arise from differential nuclear spin interactions. Furthermore, we focus on the low sensitivity nuclei ^6^Li (nuclear spin *I* = 1), and ^29^Si (*I* = 1/2), where polarization transfer via spin diffusion is expected to be limited, in order to highlight the contribution of direct polarization to the DNP enhancement. Gadolinium(III) (electron spin *S* = 7/2) was chosen as polarizing agent due to its long electron relaxation time, a fundamental requirement for polarizing agents. In addition, due to similar ionic radii, we expect it to replace Ca(II) in the structure without leading to long-range structural distortions. In agreement with previous reports, we observe a considerably lower enhancement in the amorphous sample, with a reduction in enhancement by up to a factor of 30 compared to the crystalline analogues. This raises the fundamental question of the origin of the large discrepancy. To address this, we perform careful analysis of the electron spin properties obtained from electron paramagnetic resonance (EPR) spectroscopy at various magnetic fields and correlate these findings to the NMR relaxation behavior. Our experimental results indicate shorter electron relaxation times in the glass as compared to the crystal. Short electron relaxation inhibits efficient DNP processes, thus, in line with the observed trend. The presence of a larger number of relaxation sinks^27^ in the glass, as suggested by the shorter nuclear relaxation times in the undoped sample, will as well contribute to a diminished DNP efficiency. The measured differences in magnetic resonance properties, however, do not seem large enough to entirely justify the large disparity in enhancement factors. In addition, we observe significantly larger heating in the glass sample upon microwave irradiation. This effect is attributed to a larger loss tangent value, known to be detrimental to the DNP performance.

## 2 Methods

### 2.1 Sample Preparation

For the synthesis of amorphous Li_2-x_O·Ca_1–*x*_Gd_*x*_O·2SiO_2_ glass stoichiometric amounts of the precursors, Li_2_CO_3_ (99.998% Acros Agro), SiO_2_ (99.99%, Sigma-Aldrich), CaCO_3_ (99.99% Acros Agro), and Gd_2_O_3_ (99.99% Acros Agro) were ground together for 10 min to ensure homogeneity. The mixture was placed in a platinum crucible, heated to 600 °C for 4 h for decarbonization, and subsequently molten at 1170 °C for 3 h. Finally, the samples were quenched by placing the bottom of the platinum crucible in water. To ensure a homogeneous distribution of the Gd(III) dopants, the melting procedure was done twice. Four different samples with dopant mole fractions of *x* = 0, 0.000 9, 0.001 9, and 0.003 8 were synthesized (corresponding to 19, 38, and 76 mM, respectively, assuming a constant glass density of 2.54 g/cm^3^).^28^

The same precursors were used for making the crystalline phase Li_2_CaSiO_4_. Gadolinium was doped into the crystal to have a stoichiometry of Li_2–*x*_Ca_1–*x*_Gd_*x*_SiO_4_ with *x* = 0.0015, 0.0031, 0.0061. These stoichiometries correspond to 19, 38, and 76 mM, respectively. Stoichiometric quantities of the precursors were ground for 10 min and decarbonized at 600 °C for 4 h. The powders were heated to 850 °C for 6 h and cooled to room temperature at a cooling rate of 5 °C/min.^29,30^

### 2.2 Powder XRD

X-ray diffraction measurements were performed on a TTRAX-III Rigaku diffractometer equipped with a rotating Cu anode. The X-ray (Cu Kα radiation) tube voltages and the current were 50 kV and 200 mA, respectively. The measurement range of 2θ was from 10° to 120°, with a scan rate of 2° per minute. Quantification of the phases and analysis of the crystal structure parameters were performed using the JADE 2010 software. The open angles of the divergence and scattering slits were both 0.51, and the width of the receiving slit was 0.15 mm.

### 2.3 Electron Microscopy Measurements

High-resolution scanning transmission electron microscopy (STEM) images and analytical data were recorded in a double aberration-corrected Themis Z microscope (Thermo Fisher Scientific Electron Microscopy Solutions, Hillsboro, USA) equipped with a high-brightness FEG at an accelerating voltage of 200 kV. High-angle-annular dark-field (HAADF) STEM images were recorded with a Fischione model 3000 detector with a semiconvergence angle of 30 mrad, a probe current of typically 50 pA, and an inner collection angle of 70.0 mrad. Energy dispersive X-ray spectroscopy (EDS) hyperspectral data were obtained with a Super-X G2 four-segment SDD detector with a probe semiconvergence angle of 21 mrad, a beam current of approximately 100 pA. The EDS hyperspectral data were quantified with the Velox software (Thermo Fisher Scientific Electron Microscopy Solutions, Hillsboro, USA), through background subtraction and spectrum deconvolution. Prior to measurement, samples were prepared by drop cast preparation on copper grids on ultrathin carbon foil on lacy carbon.

### 2.4 Electron Paramagnetic Resonance Measurements

Electron paramagnetic resonance (EPR) measurements were performed at three different microwave irradiation frequencies, 35 (Q-band), 120 (G-band), and 240 GHz (J-band). Q-band measurements were performed on a Bruker ELEXYS E-580 spectrometer fitted with a Q-band resonator (EN-5107-D2). An Oxford Instrument CF935 continuous flow cryostat using helium was used to control the temperature. Field sweep echo detected (FSED) spectra were acquired at 10 K. The FSED EPR spectra were recorded using the two-pulse echo sequence (π/2−τ–π–τ–echo) in which the echo intensity was measured as a function of the magnetic field. The microwave pulse lengths of π/2 and π were 10 and 20 ns, respectively. Longitudinal and transverse relaxation times at Q-band were measured with the inversion recovery experiment and the Hahn echo pulse sequence with varying echo delay, respectively.

A high frequency instrument available at the National High Magnetic Field Laboratory was used to measure electron relaxation times and FSED spectra at 120 and 240 GHz at variable temperature for both the 19 mM Gd doped glass and crystal samples. At both frequencies the data were collected on a quasi-optical spectrometer, as described in a previous work,^31^ in an arrangement without resonating structure.^32^ Unlike as described in ref (31), the 4.2 GHz intermediate frequency signal from the primary mixer is down-converted in a IQ mixing scheme using a phase-stable reference that is generated from the base frequencies of the 240 GHz source and 235.8 GHz reference oscillator. The typical pulse lengths used for the pulsed experiments are 300 ns. Longitudinal and transverse relaxation times at G- and J-band were measured with the saturation recovery experiment and the Hahn echo pulse sequence with varying echo delay, respectively.

All electron relaxation times were obtained after fitting the experimental data to stretched exponential functions (vide infra). Simulations of the EPR spectra were done in MATLAB with the EASYSPIN toolbox.^33^

### 2.5 Nuclear Magnetic Resonance Measurements

Solid-state MAS DNP experiments were carried out on a Bruker 9.4 T Avance Neo spectrometer equipped with a sweep coil and a 263.601 GHz gyrotron system. A 3.2 mm triple resonance low temperature (LT) DNP probe was used, and the experiments were performed at approximately 100 K and a MAS speed of either 9 or 10 kHz, unless specifically stated otherwise. The ^6^Li MAS NMR single pulse experiments were performed with a pulse length of 3.5 μs. To avoid background signal, ^29^Si spectra were acquired with a Hahn echo with π/2 and π pulse lengths of 3 and 6 μs, respectively. *T*_1_ relaxation and DNP buildup times were measured using the saturation recovery pulse sequence.^34^ The obtained buildup curves were fitted to a stretched exponential function according to
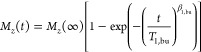
1where *T*_1,bu_ is the longitudinal relaxation or buildup time and β_1,bu_ the corresponding stretched exponent factor.

Transverse relaxation decays were measured with the CPMG^35,36^ pulse sequence for ^29^Si and with the Hahn echo pulse sequence with varying echo delays for ^6^Li. In some cases transverse relaxation times were obtained directly from the free induction decay (FID). The measured decays were fit to a stretched exponential decay function:
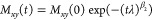
2where λ is the decay rate constant^37^ and β_2_ the stretched exponent. When the decay time λ^–1^ differs from the transverse relaxation time *T*_2_, it corresponds to either *T*_2_′ (in case of Hahn echo or CPMG measurement) or *T*_2_* (for FID). The fits of some of the FIDs required an additional oscillating term to account for off-resonance acquisition. In the solid state the decay of nuclear coherence lifetimes often has various contributions, both coherent and incoherent, which in some cases can be difficult to discern. In glasses, the NMR line shape is broadened by a distribution of isotropic chemical shifts, leading to a Gaussian shaped signal. This mechanism dominates the coherence lifetimes in one-pulse experiments of ^29^Si in all glass compositions and of ^6^Li in the weakly doped glasses. Fits of the FIDs of these samples lead to β_2_ approaching 2, a Gaussian decay. This inhomogeneous broadening is refocused by π-pulses; therefore, the decay rate constant λ becomes smaller in the Hahn echo and CPMG experiments, in the cases where inhomogeneous broadening is the dominant decay mechanism of the FID. Upon doping, the paramagnetic relaxation enhancement (PRE) effect rapidly becomes the main source of decoherence in the Hahn echo and CPMG experiments and in the FID of the crystalline samples. In these cases λ^–1^ approaches *T*_2_. Furthermore, the distribution of distances from nuclei to the paramagnetic center leads, in the absence of efficient spin diffusion, to a distribution of relaxation times, which results in decay curves with β_2_ approaching 0.5.^38^ The Fourier transform of this stretched exponential decay has no analytical solution, to our knowledge, but numerically leads to a spectrum with a “stretched Lorentzian” shape. Further discussion and justification on the used measurement to extract the transverse relaxation parameter (*T*_2_ and β_2_) as well as experimental details and fits can be found in the Supporting Information. All error estimates from the fits are given as one standard deviation.

Reported signal intensities and enhancement factors were obtained upon integration over the entire line shape. Chemical shift referencing of ^6,7^Li was performed using Li_2_CO_3_ as secondary reference, at 0 ppm at room temperature,^39^ and ^29^Si was referenced to kaolinite at −91.5 ppm.^40^ All shown spectra and transverse relaxation decays were measured following a train of saturation pulses and a recycle delay equivalent to 5 times the longitudinal magnetization buildup times, except for the undoped samples. All DNP field sweep profiles were acquired with a recycle delay of 60 s and thus not at the steady-state condition.

DNP field sweep profiles were simulated following the approach developed by Shimon et al.^41^ This approach consists of estimating the shape of the sweep profile, *S*_SE/CE_(*B*), uniquely from the EPR spectrum, *g*_EPR_(*B*). Therefore, each point  in the EPR spectrum is treated as an uncorrelated δ function. The solid effect sweep profile is obtained by computing for each field  a positive and a negative response at  respectively, weighted by the EPR intensity,  at . Equivalently, we can write^42^
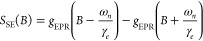
3The cross effect is obtained by additionally weighing the response by the EPR intensity at 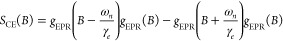
4From these equations it follows that the absolute maximum of the positive and negative lobe in the SE will be at  and  from the maximum of the EPR line, respectively, thus separated by  In the CE, on the other hand, the absolute maxima will be at a position where the product  is maximized, respectively. For a symmetric EPR line, the separation of the absolute maxima of the individual lobes will be  It is important to note that in the presence of broad EPR lines, partial cancellation of both lobes can lead to an increase in the distance of the measured maximum and minimum, in both mechanisms, SE and CE. Best fit parameters from the measured EPR spectra were used to simulate the corresponding EPR spectra at 263.601 GHz and 100 K. This approach requires the assumption that the EPR line width is dominated by inhomogeneous broadening up to this temperature range. In addition, this approach is known to be a simplification, as it does not consider the effect of the coupling network of the electron spins;^43^ nonetheless, it can assist in estimating the relative contribution of a given DNP mechanism to the overall enhancement.

## 3 Results

### 3.1 Structural Characterization

To study the influence of structural disorder on the DNP process, two samples of similar composition were synthesized: crystalline Li_2_CaSiO_4_ and glassy Li_2_OCaO·2SiO_2_. A series of samples for both structures were prepared with varying gadolinium dopant concentration of 0, 19, 38, and 76 mM. We expect Gd(III) to replace Ca(II) in the structure due to akin ionic radii^44^ and speculate that the additional charge might be neutralized by lithium ion vacancies. Preliminary characterization of the samples was done using powder X-ray diffraction (PXRD) and scanning electron microscope energy disperse X-ray spectroscopy (SEM EDX).

#### 3.1.1 X-ray Powder Diffraction and STEM

**Figure 1:**
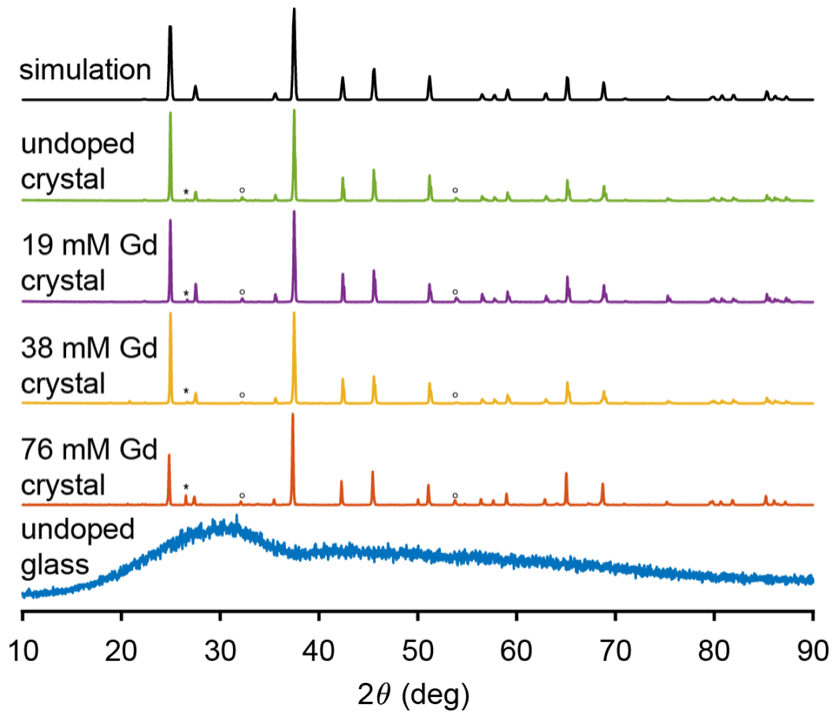
X-ray powder diffraction patterns of the undoped Li_2_OCaO·2SiO_2_ glass and all Li_2_CaSiO_4_ crystal samples, together with the reference spectra obtained from the literature.^30^ The major impurity peaks are attributed to Li_2_O and SiO_2_ phases and are indicated with asterisks and circles, respectively.

The diffraction patterns of the crystalline undoped and Gd(III) doped Li_2_CaSiO_4_ samples are shown in Figure 1. Phase analysis confirmed the formation of the expected *I*4̅2*m* space group^30^ with phase purity above 95%. Addition of small quantities of gadolinium did not alter the lattice parameter significantly, although we note that for the highest doped sample a higher amount of Li_2_O impurity was detected. The diffraction pattern of the glass (also shown in Figure 1) is broad, as expected due to its amorphous nature. The absence of sharp peaks confirms the lack of crystalline phases in this sample, no difference was observed in the diffraction pattern of the glass samples upon doping.

**Figure 2:**
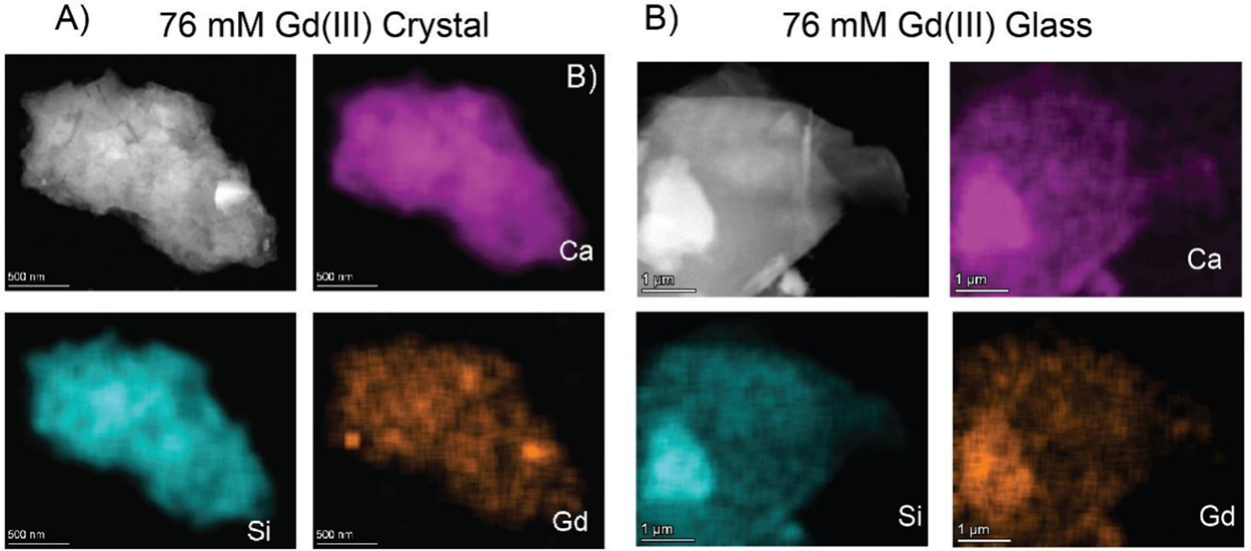
HAADF image and EDS elemental maps of (A) 76 mM Gd(III) doped crystalline Li_2_CaSiO_4_ and (B) 76 mM Gd(III) doped Li_2_OCaO·2SiO_2_ glass. The elements corresponding to the EDS are also denoted in the image. The stoichiometry determined by STEM EDS for the 76 mM Gd(III) doped glass and crystal samples is given in the Supporting Information. Gd(III) concentrations measured over multiple particles varied between 0.1% and 0.4% in both cases.

In order to assess the homogeneity of the dopant distribution in the structure, STEM EDS mapping of the 76 mM Gd doped crystalline Li_2_CaSiO_4_ and glassy Li_2_OSiO_2_*·*CaOSiO_2_ samples was done. Quantitative analysis of the gadolinium content is complicated by its low quantity; thus, we only attempted these measurements at the highest dopant concentrations. Some representative results are shown in Figure 2, and further images and quantitative analysis of the elements from the EDS spectra are given in the Supporting Information. The results confirmed the formation of phases with the expected stoichiometry within error. Mostly a homogeneous distribution of the Gd(III) dopant in the structure was found, although some particles of the crystalline sample did show the presence of gadolinium rich regions (Figure S2). No such segregation was observed for the glass sample.

#### 3.1.2 Solid State NMR Measurements

The structure of pure silica glass, SiO_2_, consists of a network of corner linked SiO_4_ tetrahedra. Addition of so-called network modifiers, such as alkali or alkaline earth ions, leads to breakage of Si–O–Si bridges and consequent depolymerization of the silicate network. The silicon sites are classified by the number of bridging oxygens *n* in the tetrahedron with the label Q^*n*^, where *n* can take values between 4 and 0.^45^ In Li_2_OCaO·2SiO_2_ the ratio of bridging to nonbridging oxygens is one to one; therefore, for a binomial distribution of Q^*n*^ sites, this system would only consist of Q^2^ species. On the other hand, in crystalline Li_2_CaSiO_4_ each element has only one unique crystallographic site. Calcium occupies a dodecahedral site with eight coordinated oxygen atoms, and the silica tetrahedra are fully depolymerized; thus only Q^0^ sites are expected.

**Figure 3:**
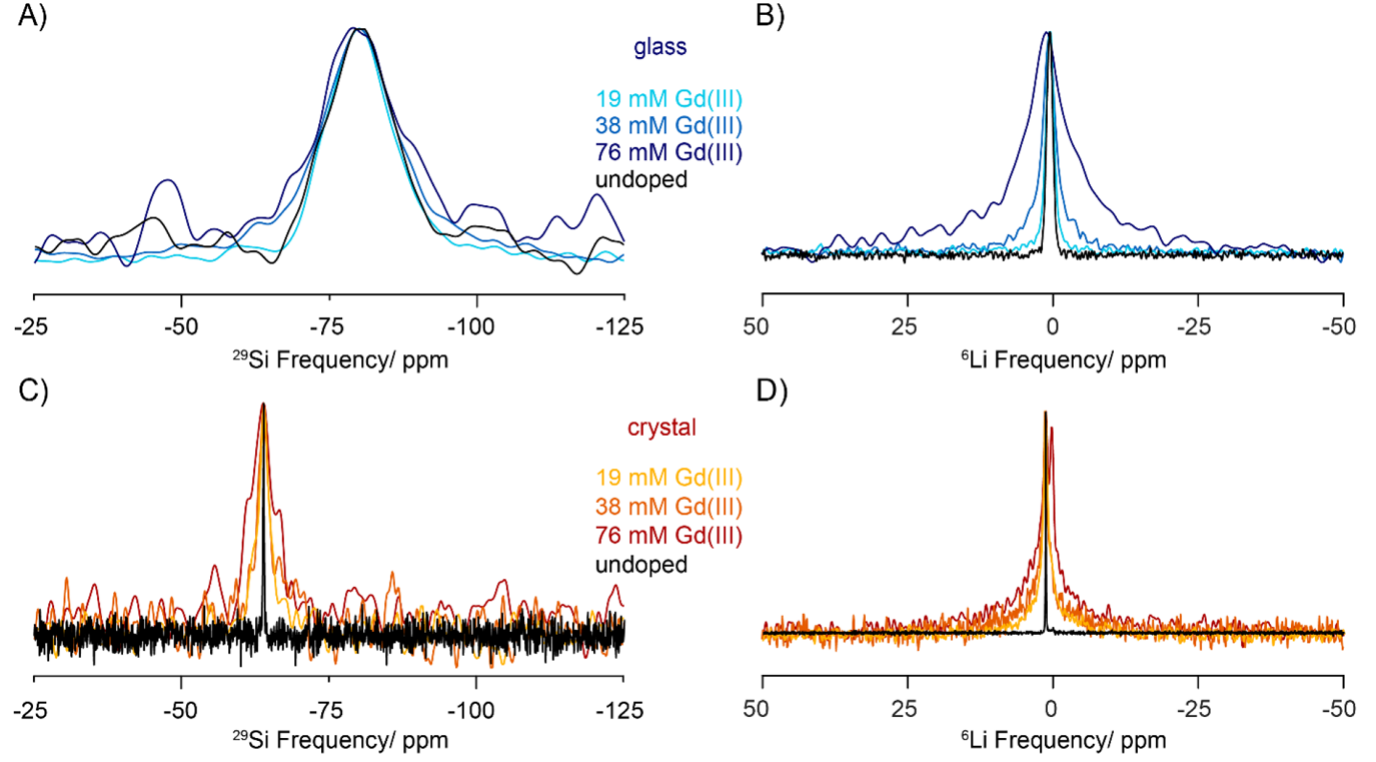
^29^Si (A, C) one-pulse and ^6^Li (B, D) Hahn echo MAS NMR spectra of amorphous Li_2_OSiO_2_·CaOSiO_2_ (top) and crystalline Li_2_CaSiO_4_ (bottom). All measurements were acquired at approximately 100 K and a spinning speed of 9 kHz. Comparison with the spectra obtained under microwave irradiation are provided in the Supporting Information.

NMR spectroscopy of ^29^Si is a particularly well suited technique for differentiating Q^*n*^ sites. The isotropic chemical shift increases stepwise with decreasing number of bridging oxygens, while Q^4^ sites usually resonate at around −110 ppm and Q^0^ sites resonate at about −65 ppm.^46^ The ^29^Si MAS NMR spectra of all samples are shown in Figure 3. The spectra of the crystalline samples show a unique silicon site at a chemical shift of −64 ppm, in good agreement with the expected range of chemical shifts for Q^0^ sites in crystalline silicates.^46^ The ^29^Si spectra of the glasses are significantly broader with a chemical shift centered at about −80 ppm, consistent with a predominant amount of Q^2^ sites.^12^ The large broadening in the glass samples with low dopant content is inhomogeneous in nature and a consequence of the disordered nature of the structure. There are various contributions to the overall line shape: on one hand, the disproportionation of Q^2^ sites to Q^1^ and Q^3^ sites, and on the other, a distribution of isotropic chemical shifts within each type of site, reflecting a distribution of bond lengths and angles encountered in glasses. The corresponding ^6^Li spectra are also shown in Figure 3. In most cases, a single peak is observed, centered at a chemical shift of 1.4 ppm for the crystal and 0.5 ppm for the glass. However, we also note the appearance of a second peak in the ^6^Li spectrum of the 76 mM doped crystalline sample at a chemical shift of 0.3 ppm and with an order of magnitude longer *T*_2_ relaxation time. In addition, unlike the peak at 1.4 ppm, this peak does not get enhanced by DNP (Figure S5). We attribute this peak to the presence of impurities, while its precise nature is not clear. The X-ray powder diffraction pattern of this sample also revealed the presence of undesired phases; however, we had attributed those to the presence of Li_2_O, which resonates at 2.8 ppm.^47^

The most prominent effect on the NMR spectrum caused by introducing low quantities of Gd(III) is homogeneous broadening of the resonances due to shortening of the transverse relaxation times. This response is a result of the Gd(III) long electron spin relaxation time (long relative to other paramagnetic metal ions,^48^ on the order of μs; *vide infra*), as long electron relaxation times severely reduce the nuclear transverse relaxation times. Nuclei in close proximity to the paramagnetic center which could experience strong Fermi contact shifts and dipolar couplings are most likely quenched in our measurements due to short *T*_2_ relaxation times and thus do not contribute to the measured line shape.^49^ The distribution of distances to the paramagnetic center leads to a distribution of relaxation times and consequently a stretched exponential decay of the transverse magnetization. Thus, when the free induction decay (FID) is governed by *T*_2_ this leads to a spectrum with a “stretched Lorentzian” shape, as observed in the doped crystal samples and the ^6^Li spectra of the highly doped glass. Even in the strongly inhomogeneously broadened spectra of ^29^Si in the glass sample, a deviation from a purely Gaussian line shape and the appearance of broad tails are observed upon doping (a more quantitative analysis is given in the Supporting Information).

**Figure 4:**
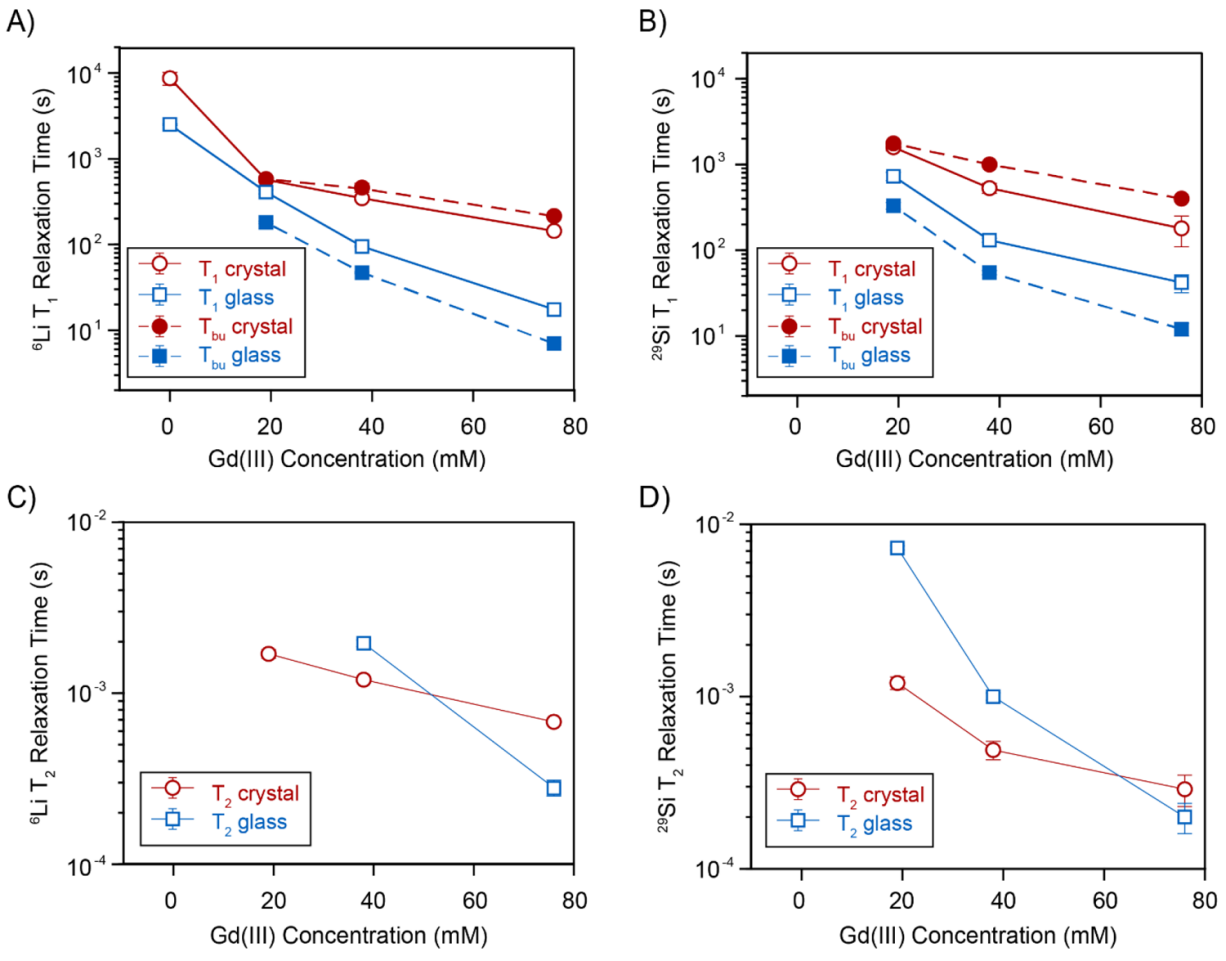
Longitudinal (A, B) and transverse (C, D) relaxation times for ^6^Li (A, C) and ^29^Si (B, D) for glass (blue squares) and crystalline (red circles) samples as a function of the Gd(III) content, obtained from best fits to eqs 1 and 2. Magnetization buildup times under microwave irradiation (*T*_bu_) are shown as filled symbols. All measurements were acquired at approximately 100 K and a spinning speed of 9 kHz. Further details on the measurements and fits are provided in the Supporting Information.

The measured ^29^Si and ^6^Li relaxation times are shown in Figure 4 as a function of paramagnetic Gd(III) content. Upon doping, the *T*_1_ relaxation times drop by at least 1 order of magnitude, compared to the undoped samples. In the undoped samples the ^29^Si saturation recovery curves have not reached a plateau after 30 000 and 12 000 s, in crystal and glass, respectively (Figure S8); therefore, the *T*_1_ relaxation times present large uncertainties. The reason for the longer relaxation time in the undoped crystal compared to the glass is likely due to the higher tendency of glasses to incorporate impurities, which, if paramagnetic in nature, can act as relaxation sources. We observe that the crystalline samples present longer *T*_1_ times over the entire concentration range. The measured relaxation times and spectra indicate that the PRE effect from the introduced Gd(III) is the main source of relaxation in all doped samples. The requirement of a stretched factor β_1_ lower than 1 for good fits (see Supporting Information) indicates a distribution of relaxation times, most likely reflecting a distribution of distances to the paramagnetic center, as expected in the absence of efficient spin diffusion.^38,50^

The NMR relaxation behavior as a function of the dopant concentration can be used to assess the homogeneity of the dopant in the sample.^51−54^ In the glass we see a nearly inverse squared dependence of the *T*_1_ and *T*_2_ relaxation times with the Gd(III) concentration as one would expect for homogeneous doping in the absence of spin diffusion.^55^ On the other hand, a significant weaker, shortening of *T*_1_ with dopant concentration is observed for ^29^Si and ^6^Li in the crystalline system, with approximately *T*_1_ ∝ [Gd]^−1.5^ and [Gd]^−1^, respectively. While in principle this could be an indication that spin diffusion homogenizes the relaxation behavior throughout the sample,^56,57^ the stretched exponential behavior of the relaxation curves (at least in the ^29^Si case) as well as the fact that the relaxation times are actually longer than in the glass do oppose this interpretation. Instead, we speculate that the observed trend is a consequence of the formation of segregated gadolinium rich phases as suggested also by the SEM EDX mapping.

In a recent work we have shown that the ratio between longitudinal and transverse nuclear relaxation times can give a first indication on whether a metal ion dopant will be suitable for DNP.^49^ This analysis requires that both relaxation processes are governed by the PRE effect. By computing the ratio of *T*_1_/*T*_2_, it is then possible to obtain a direct estimate of the correlation time describing the fluctuations of the electron magnetic moment, τ_1e_, which is a critical parameter for the success of a DNP experiment. In addition, we showed that for low concentration of dopant, τ_1e_ is a good measure of the electron relaxation time T_1e_. The correlation time τ_1e_ is simply related to the nuclear relaxation times and the nuclear Larmor frequency ω_n_ according to^49,58^
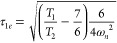
5Using this equation, we estimate τ_1e_ values using the nuclear relaxation times of ^29^Si and ^6^Li for all concentrations to be approximately 0.8 ± 0.2 μs in the glass and about 2.0 ± 0.5 μs in the crystal (see Table S9 and accompanying discussion in the Supporting Information). Thus, the crystal has about a factor of 2–3 times longer electron relaxation times.

#### 3.1.3 EPR Measurements

**Figure 5:**
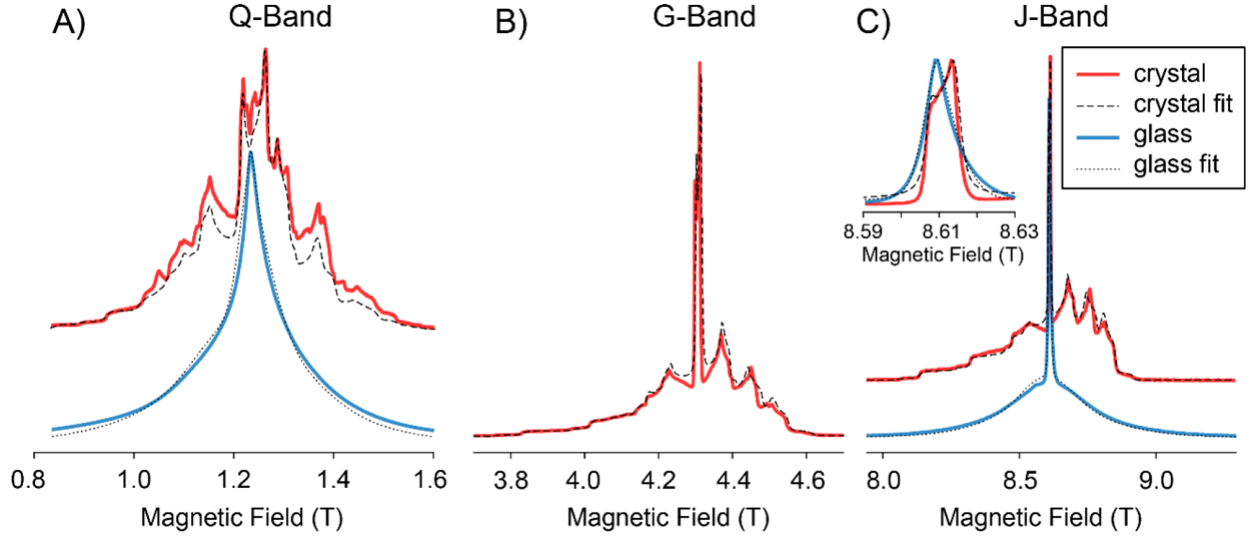
Experimental field sweep echo detected EPR spectra obtained at 10 K of Li_2_CaSiO_4_ crystal (red) and Li_2_OSiO_2_·CaOSiO_2_ glass (blue) doped with 19 mM Gd(III) at microwave irradiation frequencies of 35 (A), 120 (B), and 240 GHz (C). Corresponding simulations are shown as dashed and dotted black lines. ZFS and line broadening parameters are the same at all irradiation frequencies. Good fits of the crystalline sample required the use of two distinct Gd(III) sites of relative intensities 4:1 and with ZFS parameters of *B*_20_ = −700 MHz, *B*_22_ = 80 MHz, *B*_40_ = −0.4 MHz, and *B*_42_ = 0.4 MHz for the first site and *B*_20_ = −820 MHz, *B*_22_ = 150 MHz, and *B*_40_ = −0.5 MHz for the second. Additionally, the same magnitude of strain *D*_strain_ = 100 was added for both sites. Fitting of the glass EPR spectrum was done with a single site, with *D* = 2100 MHz and *E* = 100 MHz, with *D*_strain_ = 2000 MHz and *E*_strain_ = 2000 MHz. A *g*-value of approximately 1.992 was found for both glass and crystal samples for the measurements at 120 and 240 GHz and required a small shift for the measurement at 35 GHz, probably due to experimental inaccuracies in field and frequency determination. The relative intensity of central to satellite transitions is strongly temperature dependent, and good agreement of the fits required using temperatures of 14 and 20 K in the simulations of the crystal and glass spectra.

EPR spectra of glass and crystal samples doped with 19 mM Gd(III) measured at 10 K and various different microwave frequencies are shown in Figure 5. Best fit parameters are given in the figure caption. Fits of the crystalline spectra required higher-order zero field splitting (ZFS) terms using the extended Stevens operators.^59^ The shape of the spectra in glass and crystal is dominated by the strong zero field splitting, estimated to be larger than 2000 MHz from the fits. As the central transition (±1/2 → ∓1/2) is only affected by the ZFS to second order, its width decreases linearly with increasing field, and at the high field measurements it can be easily distinguished from the satellite transitions. The main difference between the crystal and the glass is that in the former the powder pattern of all transitions are well resolved, while in the latter the expected distribution of interaction strengths leads to a very large strain.^60,61^ Nonetheless, the spectra of both samples present very similar width and position. The high resolution of the crystalline EPR spectrum evidenced the presence of a second site with slightly larger ZFS parameter (see also Supporting Information). We speculate that the second site is related to the presence of lithium vacancies in the coordination shell of the paramagnetic dopant resulting in a reduced local symmetry. It is not possible to assess the presence of an analogous distinct site in the glass sample, as the required resolution is blurred out by the large strain.

**Figure 6:**
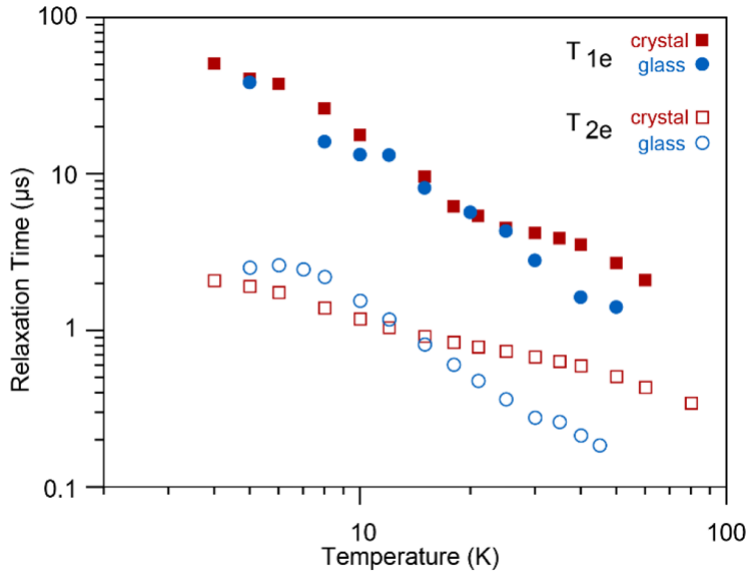
Longitudinal (filled symbols) and transverse (empty symbols) relaxation times *T*_1e_ and *T*_2e_ of glass (blue circles) and crystal (red squares) doped with 19 mM Gd(III) and measured at a microwave irradiation frequency of 240 GHz, obtained from best fits to eqs 1 and 2. Stretched factors β_1e_ of around 0.6 and 0.7 and β_2e_ of around 1.0 and 1.1 were obtained for crystal and glass, respectively, and are given in detail in the Supporting Information.

Electron relaxation times *T*_1e_ and *T*_2e_ were measured at various fields and temperatures and are given in Tables S10–S12, and the J-band results of the samples doped with 19 mM Gd(III) are summarized in Figure 6. The relaxation times decrease with increasing temperature. At temperatures below 20 K, we found mostly slightly longer relaxation times for the glass as compared to the crystal (also at Q- and G-band; see Supporting Information), but at temperatures above 20 K this trend inverts and the glass sample has increasingly shorter relaxation times compared to the crystalline sample. While knowing the properties of the electron spins at 100 K would be most interesting for the interpretation of the DNP results, it was not possible to directly measure electron relaxation times at such high temperature as they become too short. However, by extrapolating the curves, one could predict a difference in relaxation times between glass and crystal of up to about factor 3, in decent agreement with what was determined via NMR relaxation, both in terms of absolute and relative magnitude. In addition, electron relaxation times were measured as a function of the Gd(III) concentration in Q-band and at 10 K and we observed a significant decrease of *T*_1e_ and *T*_2e_ with increasing dopant content (see Table S11). Most significant, we measured a decrease in *T*_1e_ of the glass by 1 order of magnitude between 19 and 76 mM Gd(III) concentration. This steep decrease was not observed when estimating electron relaxation from τ_1e_ via NMR relaxation. Also in previous studies we only observed a very moderate decrease of *T*_1e_ with concentration within this range.^49,62^ A possible explanation for this pronounced trend could be that extrapolation of the relaxation data obtained at 10 K at Q-band frequencies is not representative of the behavior at 100 K and 9.4 T, as different relaxation mechanisms might be involved.^63^

### 3.2 Dynamic Nuclear Polarization

#### 3.2.1 DNP Sweep Profiles

**Figure 7:**
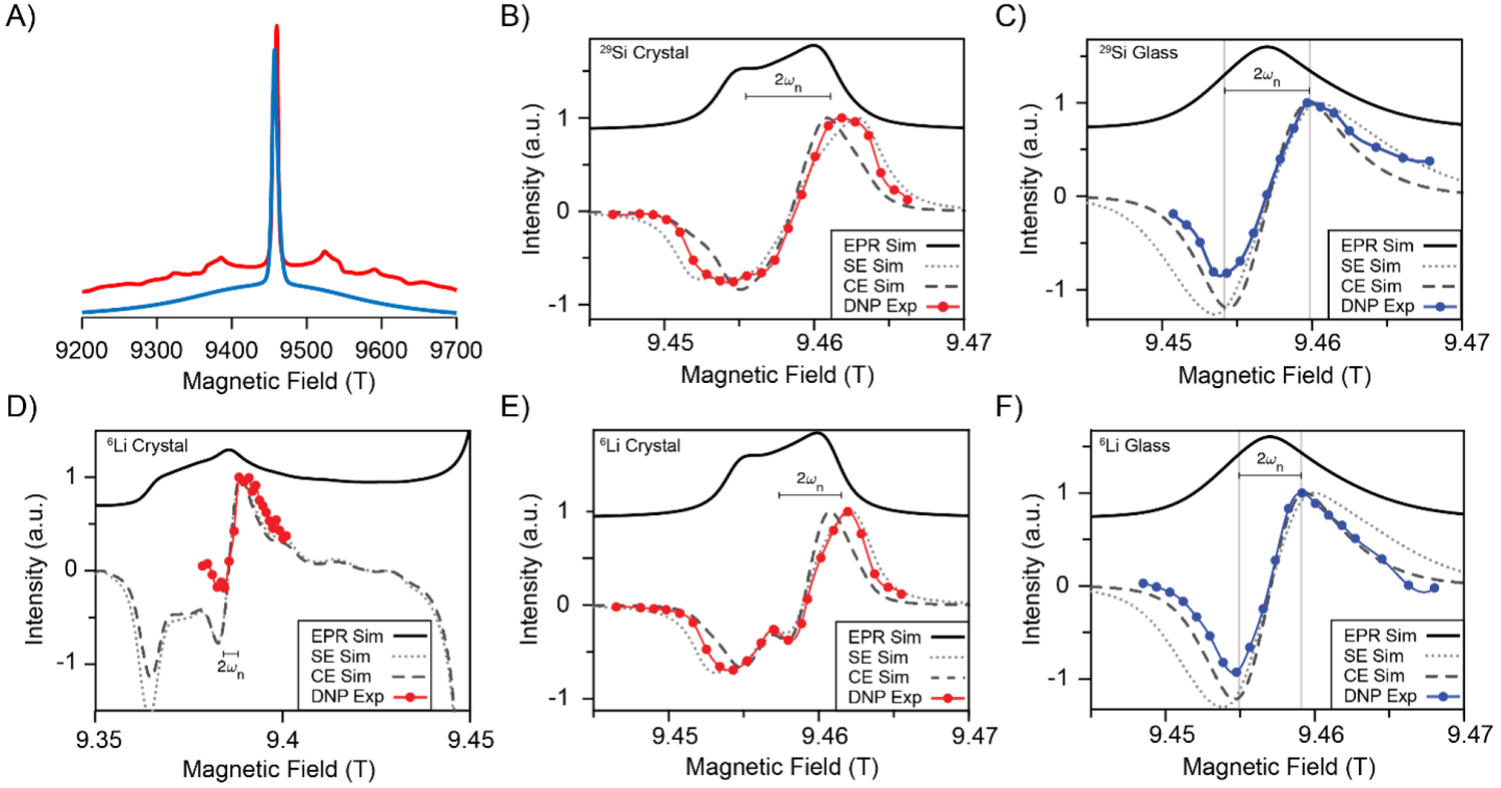
(A) Simulated EPR spectrum for a microwave irradiation frequency of 263.601 GHz at 100 K using the best fit parameter given in Figure 5 for the glass (blue) and crystal (red) samples. In (B)–(F) the ^29^Si (B, C) and ^6^Li (D, E, F) DNP field sweep of 19 mM doped crystal (B, D, E) and glass (C and F) samples are shown. Each experimental point represents the integrated signal intensity, normalized to the maximum value. Included in the figures are the simulated EPR spectrum (solid black line) and the corresponding simulation of the DNP sweep profile of the solid effect (dotted lines) and cross effect (dashed lines). The position of the field in (D) corresponds to the inner satellite transition, while all others correspond to the central transition. The DNP sweep profiles were acquired at 100 K with a spinning speed of 9 kHz. The EPR spectra were simulated using the EASYSPIN package,^33^ whereas the DNP sweep profiles were simulated using a home written MATLAB code, parting from the simulated EPR spectrum and assuming it is composed of ideal δ spin packages, according to eqs 3 and 4.^41^

The ^6^Li and ^29^Si DNP sweep profiles of the samples doped with 19 mM Gd(III) were acquired by sweeping the field around 9.4 T using a fixed microwave frequency of 263.601 GHz (Figure 7B–F). Comparing the obtained sweep profiles for the crystalline material (parts B and E) with the simulated EPR line (Figure 7A) reveals that the sweep clearly extends to frequencies beyond the limits of the EPR line of the central transition, which is a good indication that the SE mechanism is favored over the CE. Good agreement is obtained between the experimental sweeps and the SE simulation. This includes the small feature at the center of the ^6^Li sweep, which arises from the shape of the powder pattern and is obscured in the ^29^Si sweep due to its larger gyromagnetic ratio. In the sweep of the glass sample (Figure 7C,F) the tail of the lobes in the sweep profile appears to be narrower than what would be expected from the SE mechanism, although maxima and minima are to a good approximation separated by 2ω_n_.

To further investigate the possibility of the presence of the cross effect mechanism, we measured the ^6^Li DNP sweep profiles of the higher doped (76 mM) samples (Figure S11) and performed variable speed measurements on the glass sample doped with 38 mM Gd(III) (Figure S6). The shape of the sweeps at high concentrations is very similar to the less doped samples, with a slight additional broadening which we attribute to homogeneous broadening of the EPR line itself due to larger electron interactions (Figure S12). In any case, we do not see a narrowing of the separation between minimum and maximum, as was observed by Paterson et al. in an analogue analysis of zinc phosphate glasses doped with Gd(III).^17^ The measured signal enhancement is independent of the spinning speed within the measured MAS rates between 1 and 9 kHz. Both these findings point toward the absence of significant enhancement via the CE mechanism, and a broader discussion to rationalize this behavior will be given in the following section.

Remarkably, the field sweep profile around the main singularity of the powder pattern of one of the inner satellite transitions (+1/2 ↔ +3/2) shows DNP enhancement. Although the largest enhancement at the steady state condition is only 1.5 at this position, this is, to the best of our knowledge, the first report of enhancement originating from a satellite transition. This shows that DNP is possible even from an inhomogeneously broadened line spanning as much as 6.2 GHz (23 700 ppm). From this, one could envision the possibility of efficient cross effect DNP even within a single crystallite in the context of MIDNP, where the requirement becomes that the gap between the central transition and the inner satellites matches the relevant nuclear Larmor frequency.

#### 3.2.2 DNP Enhancements and Buildups

**Figure 8:**
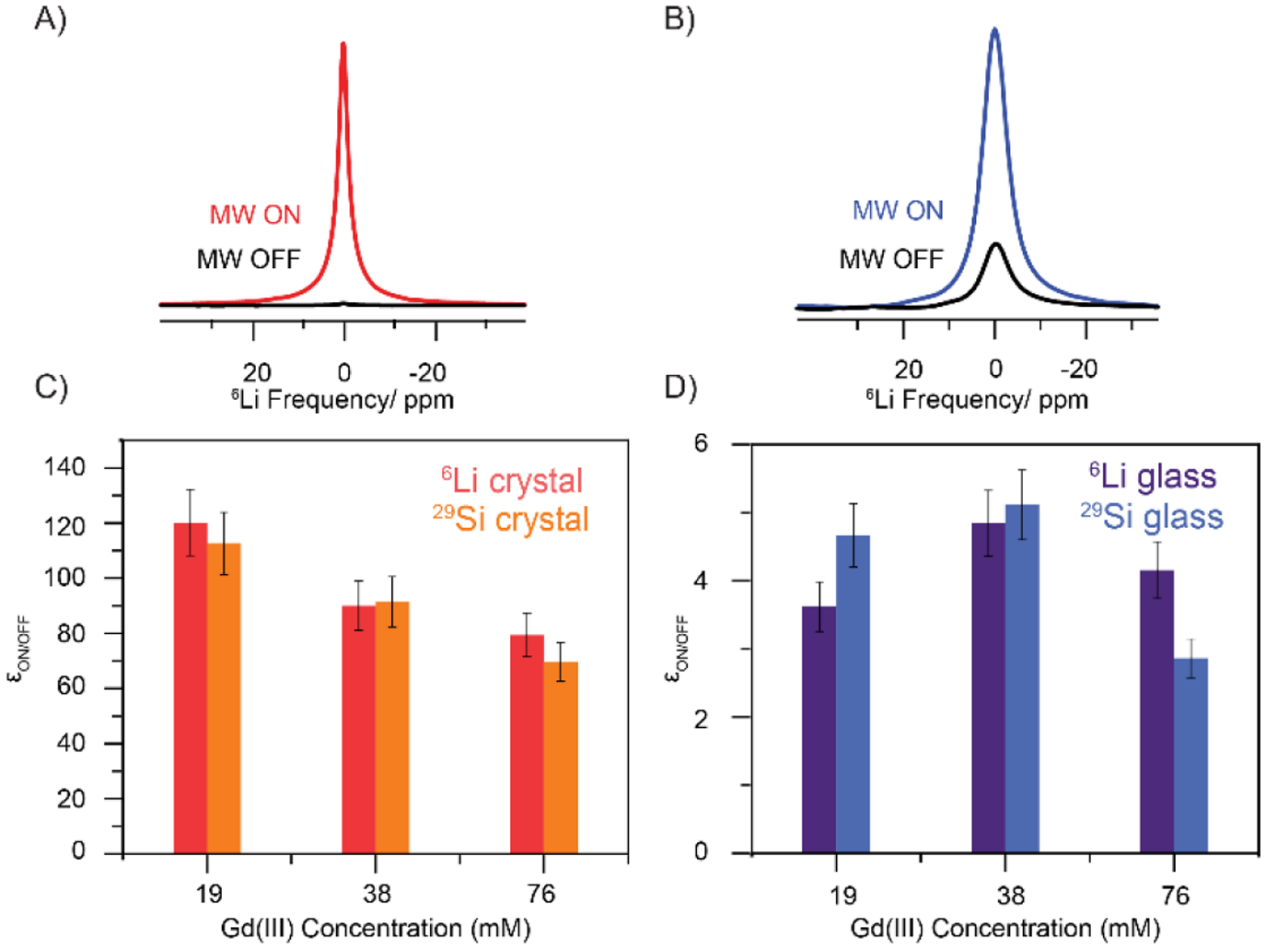
(A) and (B) show a comparison of the ^6^Li MAS NMR spectra acquired with and without microwave irradiation of the crystal and glass samples, respectively. (C) and (D) show the MAS DNP signal enhancement, as a function of the Gd(III) concentration, obtained at the optimal field position and at steady state condition for the ^6^Li (darker colors) and ^29^Si (brighter colors) of the crystal and glass samples, respectively. All measurements were done at approximately 100 K and a spinning speed of 9 kHz.

The DNP enhancements, ε_ON/OFF,_ for all compositions for ^6^Li and ^29^Si NMR were measured after optimizing the magnetic field position, corresponding to the maximum signal enhancement in the sweep profiles. The enhancements are shown in Figure 8. The crystalline samples present over an order of magnitude higher enhancements than the glasses. These results are in line with previously observed lower enhancements in amorphous oxides compared to crystalline oxides. The crystalline series shows a clear trend toward lower signal enhancements ε_ON/OFF_ with increasing Gd(III) content, from up to 120 at the lowest doping level down to 70, while in the glass samples the enhancements remain constant at around 4. Thus, the largest difference in DNP efficiency between glass and crystal was observed for a Gd(III) content of 19 mM with a factor of approximately 30.

The polarization buildup behavior for all samples was also measured under microwave irradiation. The obtained buildup times, *T*_bu_, are shown in Figure 4 together with the respective *T*_1_ relaxation times. For the Li_2_OSiO_2_*·*CaOSiO_2_ glass samples *T*_bu_ is significantly shorter for both ^29^Si and ^6^Li and at all measured concentrations. We attribute this difference to a strong heating effect of the microwaves in the glass sample (see below), leading to a shortening of *T*_1e_ and consequently shortening of *T*_1_ according to the PRE mechanism (note that  with  the nuclear Larmor frequency). This behavior could also originate from differential enhancement as a function of the proximity to the polarizing agent. However, the slightly narrower lines observed with microwaves (Figure S5) support the interpretation of significant sample heating. On the other hand, in the case of crystalline Li_2_CaSiO_4_, we observe the opposite behavior with *T*_bu_ being either similar (with 19 mM Gd(III)) or longer (38 and 76 mM) than *T*_1_. This result is unexpected, and we attribute it to the presence of segregated gadolinium rich regions, as already suggested from the STEM and NMR results. If the Gd(III) content of these segregated regions is large enough, not only will the nuclear *T*_1_ relaxation time be shorter but also τ_1e_ is expected to decrease, resulting in a lower DNP efficiency in these regions. Thus, the relative contribution of the Gd(III) rich domains will be lower in a saturation recovery measurement under DNP conditions as compared to thermal conditions (without microwaves), leading to an apparent lengthening of *T*_bu_ compared to *T*_1_.

#### 3.2.3 Sample Heating upon Microwave Irradiation

**Figure 9:**
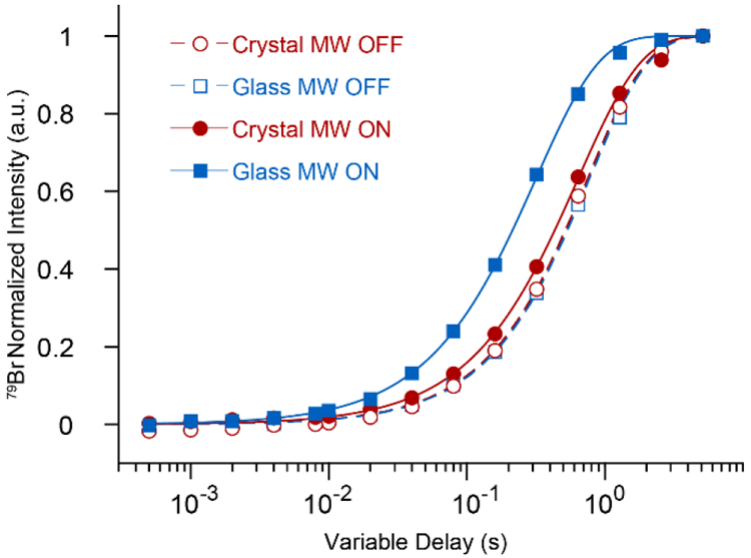
^79^Br saturation recovery buildup curves in KBr mixed in 76 mM Gd(III) doped Li_2_CaSiO_4_ crystal (red circles) and Li_2_OCaO·2SiO_2_ glass (blue squares), with a mass ratio of 1:4. Measurements were done at a spinning speed υ_R_ of 9.4 kHz with (full symbols, solid lines) and without (empty symbols, dashed lines) microwave irradiation. Curves are best fits obtained with a single exponential as shown in eq 1 with β_1_ = 1.

In an effort to rationalize the differences in enhancement and relaxation properties observed for the glass and crystal, we have also evaluated the effect of continuous microwave irradiation on the samples. Depending on the permittivity properties of the materials, this can lead to significant sample heating. In order to analyze the heating effects of microwave irradiation on both crystalline and glass materials, the changes in temperature were recorded by mixing small quantities of KBr into the sample and monitoring the ^79^Br *T*_1_ relaxation times.^64^ The results are shown in Figure 9 and clearly demonstrate a significant stronger heating effect on the silicate glass. The *T*_1_ relaxation times were reduced from 0.75 ± 0.01 s and 0.78 ± 0.01 s without microwave irradiation to 0.63 ± 0.01 s and 0.31 ± 0.01 s upon irradiation, in crystal and glass, respectively. This is equivalent to a temperature increment of 7 and 48 K, respectively.^64^

## 4 Discussion

In the previous section we showed that the DNP enhancement in amorphous silicate glasses is significantly smaller than in a crystalline sample of similar composition. The enhancement efficiency in a DNP experiment will depend on many different parameters. Here we try to assess which of these parameters is affected by the change from crystalline to amorphous nature of the sample and discuss, based on the various NMR and EPR results, how they relate to the overall efficiency.

### 4.1 Nuclear Spins

The strength of dipolar couplings in the homonuclear spin bath plays a fundamental role in the DNP process of protonated samples.^65,66^ In the case of endogenous DNP we have shown that for low sensitivity nuclei (with low natural abundance and/or gyromagnetic ratio), hyperpolarization can reach the entire sample without the requirement of spin diffusion, as long as the PRE due to the polarizing agents themselves is the main relaxation mechanism in the sample.^23^ Thus, if homonuclear couplings do not mediate relaxation, their strength should not affect the DNP performance.

We have chosen similar chemical compositions for the glass and crystal samples to ensure a similar dipolar coupling network of the nuclear spins. Nonetheless, the disordered nature of the glass leads to inhomogeneous broadening of the NMR signal, which could reduce the spin diffusion efficiency due to larger energy offsets among coupled nuclei. By focusing on the ^6^Li and ^29^Si nuclei, we expect the contribution of spin diffusion in the polarization buildups to be small, based on previous results on similar samples. Nonetheless, at the lowest Gd(III) content, the ^6^Li polarization buildup in the crystalline sample followed a simple exponential behavior, which is an indication that in this case spin diffusion is contributing to the polarization buildup. We do not, however, observe any relation between the stretch factors β_1,2_ (which are related to the efficiency of the spin diffusion process and are given in Tables S7 and S8) and the observed DNP enhancements. It appears that in this case, whether the magnetization is transferred directly from the polarizing agent to remote nuclei or via spin diffusion through intermediate nuclei does not influence the enhancement factors, as long as there are no other sources of relaxation interfering. These effects are beyond the scope of this work and will be described in detail elsewhere.

Relaxation of the nuclear spins due to mechanisms other than the PRE from the dopants will reduce the overall DNP efficiency. The shorter relaxation time measured in the undoped glass sample is indicative of additional relaxation sources, not present in the crystalline sample, most likely paramagnetic impurities. The presence of a paramagnetic center, which does not contribute as a polarizing source but causes relaxation of nearby nuclei, is known as relaxation sink and is detrimental for DNP enhancements.^27^ In the case of direct DNP, the extent of polarization transfer away from a polarizing agent is limited by the presence of alternative relaxation processes. Therefore, as a consequence of the presence of relaxation sinks, we do expect a reduced range and a lower homogeneity of the enhancements in the glass samples. While it is difficult to assess the contribution of this effect to the lower enhancements in the glass, we do emphasize that longitudinal relaxation times in the undoped samples are at least 1 order of magnitude larger compared to the doped samples, which indicates that the PRE from the polarizing agents is significantly more efficient in causing relaxation. Furthermore, no significant increment in enhancement is observed when increasing the dopant concentration in the glass samples. Based on these considerations, we expect the effect from additional relaxation sources to be small but nonetheless to have a contribution to the overall worse performance of MIDNP in the glass samples.

### 4.2 Electron Spins

In order to maximize the DNP efficiency, a homogeneous distribution of the polarizing agents throughout the sample is desired. Formation of clusters or highly doped phases can lead to a reduction or complete bleaching of the polarizing agent’s performance in those regions. This is due to enhanced dipolar couplings among the electron spins, causing line broadening and shortening relaxation times. In addition, the presence of highly concentrated regions implies that the concentration of Gd(III) in the rest of the sample will be below the nominally intended value, eventually leading to regions completely depleted of polarizing agents, where nuclear relaxation is affected by intrinsic mechanisms, also reducing the DNP enhancement of those nuclei.

Our experimental observations strongly suggest the presence of aggregation of gadolinium ions in the crystalline samples, while no such indications were found for the glass samples, pointing to a more homogeneous distribution in the latter. Thus, it is likely that the larger homogeneity achieved in the glass sample actually reduced the difference in enhancements between glass and crystal.

The EPR line shape of the polarizing agent is fundamental for the DNP process: broad lines will decrease the saturation efficiency and can lead to partial cancellation between positive and negative enhancement lobes. Comparison of the electron spin resonance of the central transition of crystal and glass reveals a very distinct shape, while a well-defined powder pattern is observed in the crystalline sample. The glass presents a Gaussian shaped signal. However, both signals have a similar full width at half-maximum (see insert of Figure 5 C). While the broad tails of the Gaussian might contribute to some extent to a larger cancellation among positive and negative lobes, it seems evident that this is not a major source of differential enhancement in this system. Furthermore, the EPR line shape will determine the accessible DNP mechanisms: the solid effect generally benefits from narrow lines. On the other hand, the cross effect mechanism requires an EPR line width at least larger than the Larmor frequency of the nucleus.

Two different mechanisms have been reported for MAS DNP using metal ions as polarizing agents: the solid effect (SE) and the cross effect (CE). The SE DNP mechanism requires a single electron coupled to a single nucleus, and enhancement can be expected whenever it is possible to saturate the formally forbidden zero- or double-quantum transitions. The cross effect, on the other hand, requires two coupled electron spins, with a nuclear spin being coupled to at least one of the electrons. The main advantage of this mechanism over the solid effect is that the DNP enhancement requires saturation of an allowed single quantum transition of one of the electrons.^67^ Therefore, the nutation frequency of the microwave irradiation will not be scaled by the nuclear Larmor frequency and the transition will be easier to saturate. Thus, making the CE accessible is highly desirable, especially when going to high magnetic fields.

A limiting factor for accomplishing the CE is ensuring the presence of two electrons sufficiently strongly coupled fulfilling the cross effect condition  when randomly distributed in the structure. In exogenous DNP this condition is enforced by using polarizing agents specifically tailored for this purpose, generally these are nitroxide biradicals.^20,68,69^ In the context of metal ions, the use of bis(Gd-chelates) has also been demonstrated to assist in obtaining CE.^70^

The appearance of the CE condition requires the presence of coupled spins from magnetically inequivalent sites. While we saw that the EPR line of crystalline and glass sample showed a similar full width at half-maximum and both are dominated by inhomogeneous broadening, there is an important and fundamental difference in the origin of the broadening. The powder pattern of the former arises from the presence of many crystallites with different orientations. Within a single crystallite paramagnetic metal ions from a given site will have the same resonance frequency independent of the rotor position. A very different scenario is found in an amorphous material, where the broadening arises from the presence of multiple environments within each particle. Since the number of spins coupled across different particles is negligible compared to the total number of spins within these micrometer sized particles, we can expect the probability of coupled spin pairs capable of ensuring the CE condition being present to be higher in the glass sample. Higher concentration of polarizing agents should benefit the CE mechanism by increasing the number of coupled spins.^41^ The absence of a significant concentration dependence, on the other hand, would be in line with the expected behavior for the SE mechanism;^23^*vide infra*.

The efficiency of the CE mechanism is known to depend on the MAS rate.^71−73^ Two main aspects, disregarding effects from depolarization,^74,75^ contribute to this dependency. First, the polarization transfer during the rotor events becomes less efficient with increasing spinning speed, as the adiabaticity of the events drops. And second, the polarization difference between the two electron spins achieved in a microwave event needs to persist until a CE rotor event; with increasing spinning speed the separation between rotor events becomes shorter. Since these two aspects have opposite MAS rate dependency, generally an increase in the signal enhancement is followed by a decrease, after passing an optimum spinning speed. In nitroxide biradicals used for DNP via the CE mechanism *T*_1e_ values in the order of milliseconds are encountered.^76,77^ Here, we have determined relaxation times of only a few microseconds, while the rotor period spinning at 10 kHz is 100 μs. Given the large difference in time scales, it is highly unlikely that nuclear polarization can build up constructively via the CE mechanism in these experiments but would require much faster spinning speeds. This is further corroborated by the absence of any effect of spinning speed on the signal enhancement, which again would agree with DNP driven by the SE mechanism.^72^

After having ruled out the presence of the CE mechanism in either system, glass or crystal, we turn our attention to the solid effect DNP mechanism. SE DNP can occur between a nucleus coupled to a single electron upon microwave irradiation on either the zero or double quantum transition. Since these transitions are formally forbidden, the effective nutation frequency  will be scaled by the strength of the dipolar coupling divided by the nuclear Larmor frequency, (78) Saturation of these transitions is generally not achieved in MIDNP and is therefore a major limiting factor of signal enhancement.^16^ The analytical expression for the saturation efficiency, assuming that the polarizing agent is at the same time the main source of relaxation, is related to *T*_1e_ and *T*_2e_ according to^23,49^
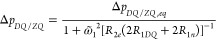
6where  is the polarization of the irradiated (DQ or ZQ) transition.  are the relaxation rates, with  and  assuming that one can replace τ_1e_ by *T*_1e_. A more detailed derivation of this relation was given in previous studies.^23,49^ The important consequence is that enhancements benefit from longer electron relaxation times (both *T*_1e_ and *T*_2e_). In principle a quadratic dependence is expected at low saturation efficiencies (assuming  with a bend toward a weaker dependence approaching higher efficiencies. In an experimental study we actually found a linear relation within a range of almost 1 order of magnitude in τ_1e_, which we attributed to this bending.^49^ From the experimentally determined differences in electron relaxation times in this study it is clear that they will contribute to the observed divergence in DNP enhancements among glass and crystalline samples. However, a reduced electron relaxation time by a factor of 2–3 does not seem sufficient to account for the observed difference in enhancement of up to factor of 30 and neither for a linear nor for a quadratic relation.

In addition, we can also use eq 6 to understand the weak concentration dependence of the signal enhancement. By looking at the various terms in the equation, it becomes evident that the enhancement is independent of the strength of the dipolar coupling as long as the nuclear relaxation is governed by the PRE. Consequently, the SE enhancement is also independent of the concentration of the polarizing agent, at least until the properties of the electron spin themselves (line width and τ_1e_) are affected by the increasing strength of the electron–electron interactions.^49^ This last point is likely a reason for the observed slight decrease in enhancement with increasing Gd(III) content in the crystalline samples.

### 4.3 Dielectric Properties

Finally, as the differences arising from the nuclear and electronic spin properties of both systems do not seem to suffice to explain the large discrepancy in signal enhancement, we turn our attention to the dielectric properties of both materials. The response of an insulating sample to the presence of an oscillating electric field is described by its dielectric properties. In an ac circuit consisting of a simple capacitor, the current will be 90° out of phase with respect to the voltage when vacuum is used as the dielectric. Deviation from this value in a dielectric will lead to a power loss, quantified by the loss tangent  where  and  are the imaginary part and real part of the dielectric constant, respectively.^79^ The loss tangent is therefore a measure of a materials capacity to dissipate the absorbed energy into heat. A large loss tangent will result in large sample heating. Therefore, useful information on a materials dielectric properties is obtained by analyzing the temperature rise upon microwave irradiation of the sample at the conditions of interest.

For DNP purposes, large loss tangent values are known to be detrimental.^80,81^ This is due to three reasons:^82^ First, the absorption of microwaves reduces the photons available for DNP purposes. Second, the heating caused by microwave absorption leads to shortening of the electron relaxation times. And third, the increased temperature will reduce the equilibrium population difference according to the Boltzmann distribution. This will lead to an apparent lower enhancement factor when comparing the signal intensity to the measurement without microwave irradiation at colder temperature.

Our experimental findings showed that the glass sample was heated significantly stronger as compared to the crystalline material, indicating a higher dielectric loss. Difference in dielectric loss could in principle arise from distinct ionic conductivity, although in the frequency region of hundreds of gigahertz, ionic motion is not expected to be a major contributor to the loss tangent.^83^ In addition, the disordered nature of the glass structure is known to lead to increased phonon scattering processes, which in turn are one of the main sources of dielectric loss in the microwave frequency range.^84^ For this reason silicate glasses are often found to have larger dielectric loss compared to their crystalline analogues.^84^ We do expect higher gains from MIDNP in glasses with lower dielectric loss, such as alkali-free silicates, including boro- and aluminosilicate glasses.^83^

## 5 Conclusions

In this study we have investigated the difference in performance of endogenous metal ions DNP between amorphous and crystalline oxides. Comparison of a silicate glass and crystal of similar chemical composition doped with Gd(III) enabled us to investigate fundamental differences in both types of materials. More than 1 order of magnitude lower enhancement is obtained in the silicate glass, compared to its crystalline analogue, independent of the dopant concentration. Analysis of the DNP response indicates that the solid effect mechanism dominates in both cases, glass and crystal. Furthermore, we are able to rule out differences in the nuclear spin bath as well as in the width of the EPR line as a major source of the discrepancy in enhancements. Instead, we find two main reasons for the reduced DNP efficiency in the glass: intrinsically shorter electron relaxation times as well as unfavorable dielectric properties arising from the disordered structure of the glass. In addition, the higher tendency to incorporate paramagnetic impurities in the glass material will introduce relaxation sinks, which further diminish the DNP efficiency. Our findings suggest that many technological relevant oxide glasses are unlikely to significantly benefit not only from the metal ions based DNP approach but from DNP in general. Finally, we demonstrate the possibility of obtaining DNP enhancements from a satellite transition of an electron spin 7/2 with large ZFS.

## Supporting Information Available

EM and EDX measurements and analysis; additional details on NMR measurements, DNP enhanced NMR spectra, and NMR relaxation analysis; DNP sweep profiles of highly doped samples; additional EPR spectra, line shape deconvolution, and electron relaxation parameter at various fields and temperatures (PDF)
