# Supplementary material for: The Effect of Disorder on Endogenous MAS-DNP: Study of Silicate Glasses and Crystals
Source: J Phys Chem C Nanomater Interfaces. 2023 Feb 27;127(9):4759–72. doi: 10.1021/acs.jpcc.2c08849 (PMC10009812; doi:10.1021/acs.jpcc.2c08849)
Supplement: Supplementary file 1 — jp2c08849_si_001.pdf [file jp2c08849_si_001.pdf]

Supporting Information for:

The Effect of Disorder on Endogenous MAS DNP: Study of Silicate Glasses and Crystals

Brijith Thomas[1#], Daniel Jardón-Álvarez[1#], Raanan Carmieli[2], Johan van Tol[3], Michal Leskes[1*]

[1]*Department of Molecular Chemistry & Materials Science, Weizmann Institute of Science Rehovot 76100, Israel.*

[2]*Department of Chemical Research Support, Weizmann Institute of Science Rehovot 76100, Israel.*

[3]*National High Magnetic Field Laboratory, Florida State University, Tallahassee, FL 32310, USA.*

[#]These authors contributed equally

*michal.leskes@weizmann.ac.il

Table of Contents

1. STEM images and EDS data 3

Case 1: Li_2CaSiO_4 crystal 3

Case 2 Li_2CaSiO_4 crystal 5

Case 3: $Li_2OCaO{\cdot}2SiO_2$ glass 6

Case 4: $Li_2OCaO{\cdot}2SiO_2$ glass 7

2. Solid State NMR 9

NMR Spectra 9

Variable MAS experiments 10

$T_{1,bu}$ Measurements 11

T_2 Relaxation 13

The T_1/T_2 ratio 17

DNP field sweeps 18

3. EPR 18

EPR Spectra and Fits 18

T_{1e} and T_{2e} Relaxation 20

1. STEM images and EDS data

STEM images of two different samples were collected, the highest doped crystal and glass compositions. Corresponding EDS data were also collected. In the following four case studies are presented, representing two particles of each sample. The EDS mapping of cases 1 and 3 is shown in the main text. The expected atomic percentages are: crystal - Ca:Si:O - 17:17:66 and glass Ca:Si:O-11:22:67

Case 1: Li_2CaSiO_4 *crystal*

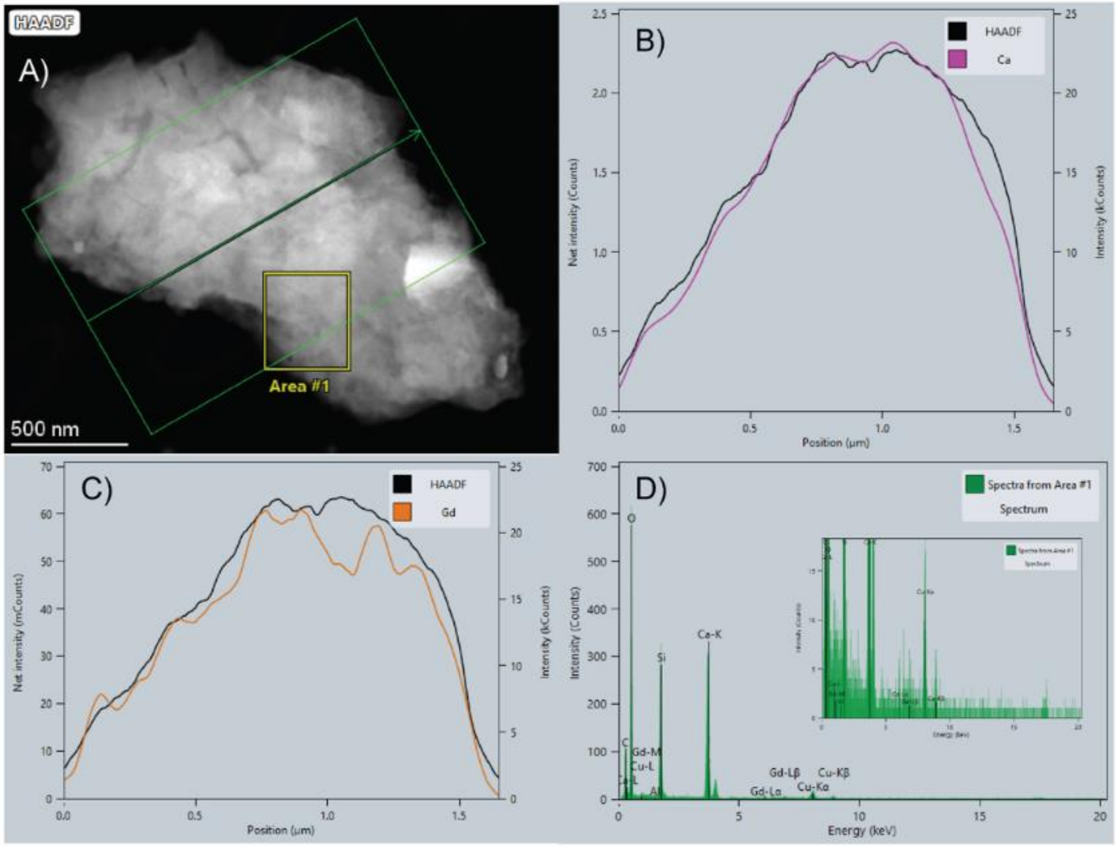

Figure S1 (A) STEM-EDS data of the 76 mM Gd(III) doped $L_{i2}CaSiO_4$. (B) and (C) show a line profile of net intensity of calcium-K and gadolinium-L X-ray lines with respect to the HAADF (high angle annular dark field) signal. Gadolinium follows the HAADF signal. The HAADF signal is a measure for the projected mass density of the volume, this indicates that gadolinium is homogeneously distributed in the volume. (D) shows the cumulated EDS spectrum of the selected area Area#1 in (A). The elemental quantification shown in the Table S1 matches the expected stochiometric composition.

Table S1: Quantification of the elements from the EDS spectra shown in Figure S1.

**Z**	**Element**	**Family**	**Atomic Fraction (%)**	**Atomic Error (%)**	**Mass Fraction (%)**	**Mass Error (%)**	**Fit Error (%)**
8	O	K	63.90	6.06	44.95	2.71	0.52
14	Si	K	17.41	3.74	21.50	4.34	0.74
20	Ca	K	18.58	3.03	32.74	4.78	0.15
64	Gd	L	0.12	0.02	0.80	0.11	6.57

Case 2 Li_2CaSiO_4 crystal

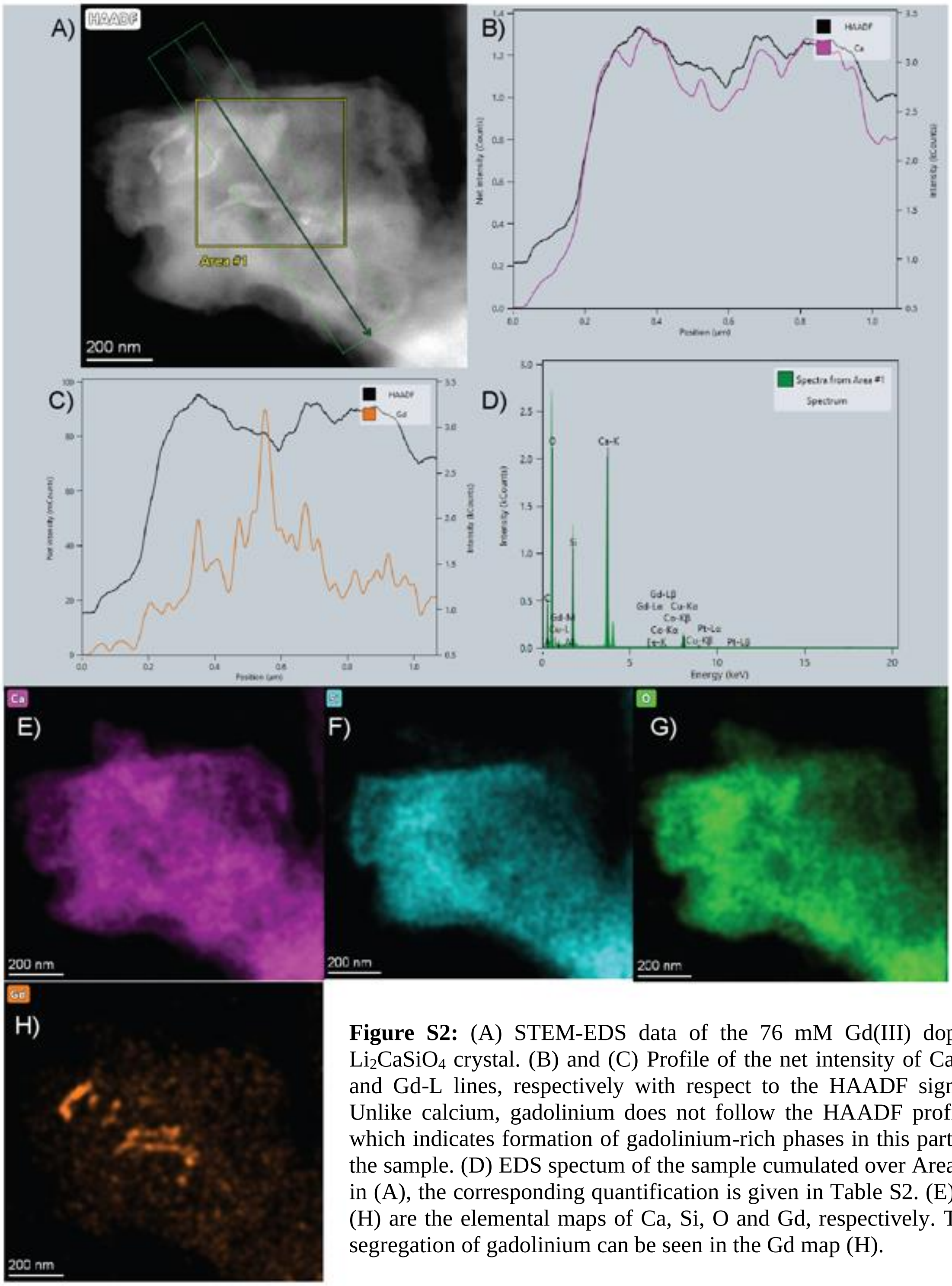

Figure S2: (A) STEM-EDS data of the 76 mM Gd(III) doped Li_2CaSiO_4 crystal. (B) and (C) Profile of the net intensity of Ca-K and Gd-L lines, respectively with respect to the HAADF signal. Unlike calcium, gadolinium does not follow the HAADF profile, which indicates formation of gadolinium-rich phases in this part of the sample. (D) EDS spectum of the sample cumulated over Area#1 in (A), the corresponding quantification is given in Table S2. (E) to (H) are the elemental maps of Ca, Si, O and Gd, respectively. The segregation of gadolinium can be seen in the Gd map (H).

Table S2: Quantification of the elements from the EDS spectra shown in Figure S2.

Z	Element	Family	Atomic Fraction (%)	Atomic Error (%)	Mass Fraction (%)	Mass Error (%)	Fit Error (%)
8	O	K	66.96	6.12	47.37	2.85	1.84
14	Si	K	12.51	2.66	15.54	3.12	0.49
20	Ca	K	20.39	3.27	36.14	5.23	0.18
64	Gd	L	0.14	0.02	0.96	0.12	1.51

Case 3: $Li_2OCaO{\cdot}2SiO_2$ glass

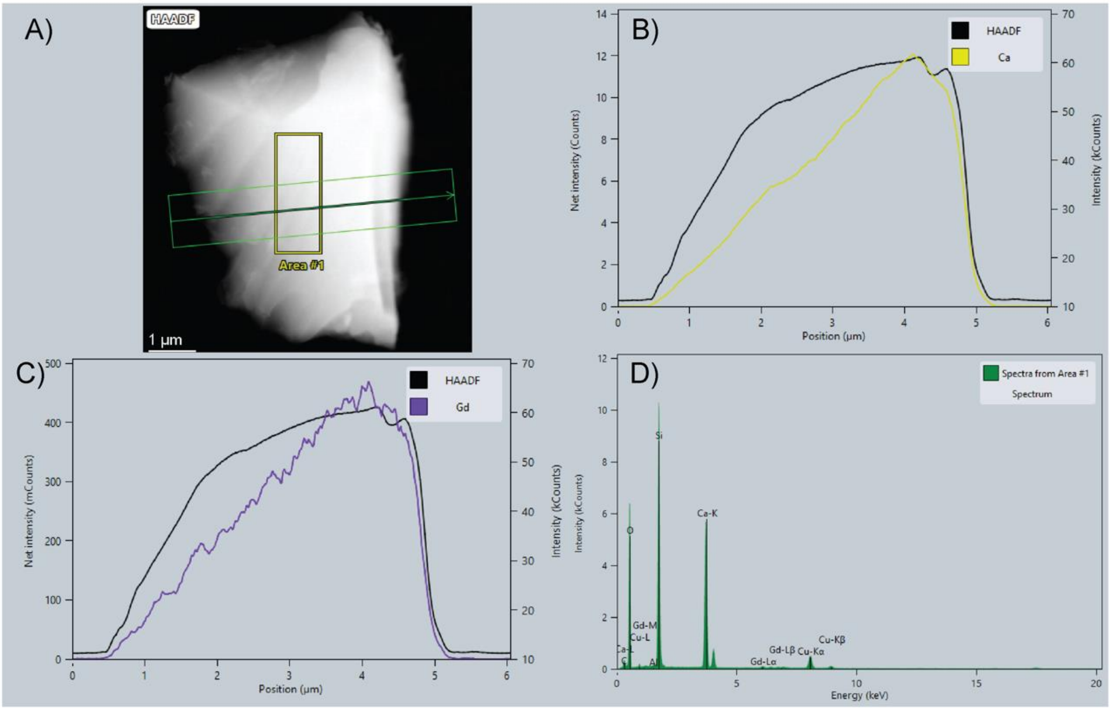

Figure S3 (A) STEM-EDS data of the 76 mM Gd(III) doped Li2OCaO·$2SiO_2$ glass. (B) and (C) Line profile of net intensity of calcium-K and gadolinium-L lines with respect to HAADF. The gadolinium profile follows the HAADF signal. The HAADF signal represents the projection of the mass density of the volume, this indicates gadolinium that is homogenously distributed in the volume. (D) EDS spectrum cumulated over the selected area Area#1 in (A). The quantification shown in Table S3 matches the expected stoichiometric composition.

Table S3: Quantification of the elements from the EDS spectra shown in Figure S3.

Z	Element	Family	Atomic Fraction (%)	Atomic Error (%)	Mass Fraction (%)	Mass Error (%)	Fit Error (%)
8	O	K	37.16	5.78	22.37	2.09	0.57
13	Al	K	0.15	0.04	0.16	0.03	3.75
14	Si	K	40.08	9.92	42.37	9.06	0.21
20	Ca	K	22.38	4.58	33.76	5.48	0.07
64	Gd	L	0.23	0.04	1.34	0.19	0.38

Case 4: $Li_2OCaO{\cdot}2SiO_2$ glass

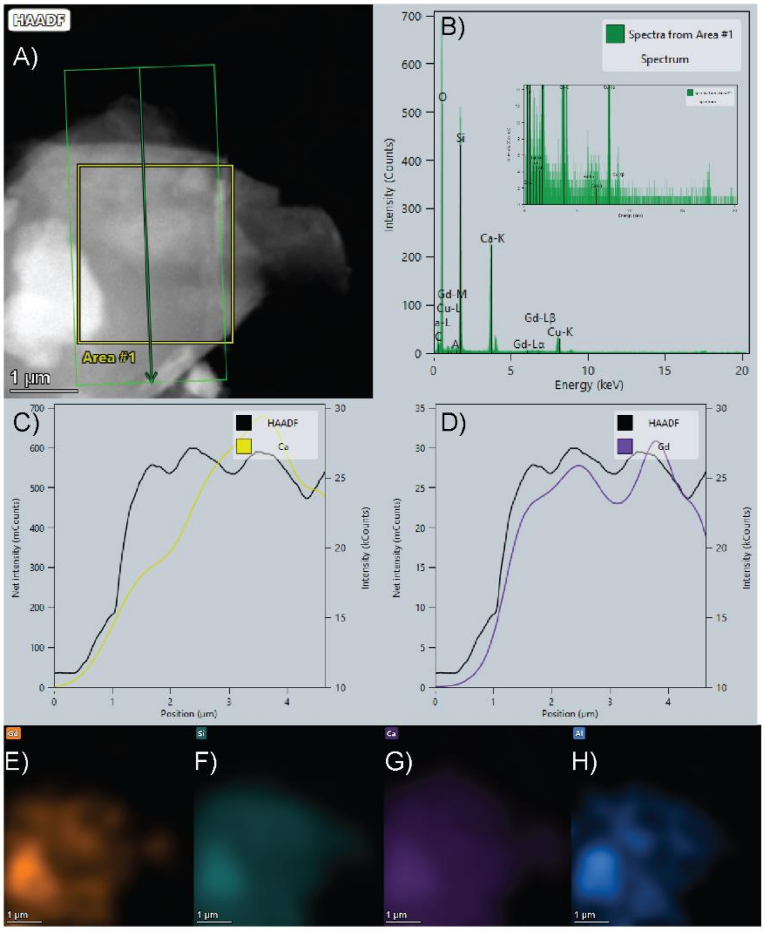

Figure S4 (A) STEM-EDS data of the 76 mM Gd(III) doped $Li_2OCaO{\cdot}2SiO_2$ glass. (B) and (C) Profile of the net intensity of Ca-K and Gd-L lines, respectively with respect to HAADF signal. The gadolinium profile follows the HAADF signal, indicating that gadolinium is homogenously distributed in the volume. (D) EDS spectrum cumulated over Area#1 in (A), the corresponding quantification is given in Table S4. (E) to (H) are elemental maps of Gd, Si, Ca and Al respectively. A small amount of aluminum also observed in the EDS spectra.

Table S4: Quantification of the elements from the EDS spectra shown in Figure S4.

Z	Element	Family	Atomic Fraction (%)	Atomic Error (%)	Mass Fraction (%)	Mass Error (%)	Fit Error (%)
13	Al	K	0.66	0.16	0.56	0.09	9.55
14	Si	K	68.69	20.40	59.86	13.80	1.52
20	Ca	K	30.25	7.92	37.62	6.89	0.30
64	Gd	L	0.40	0.10	1.96	0.34	4.80

2. Solid State NMR

NMR Spectra

Table S5: Recycle delay and number of scans, given in brackets, of the spectra shown in Figure 3 in the main document.

Gd(III) concentration	Recycle delay (s)			
	Glass ^{6}Li	Glass ^{29}Si	Crystal ^{6}Li	Crystal ^{29}Si
undoped	25000 (1)	12000 (2)	12000 (1)	30000 (2)
19 mM	1200 (2)	8000 (4)	2400 (1)	4000 (4)
38 mM	600 (4)	2000 (16)	2400 (1)	4000 (4)
76 mM	120 (8)	480 (16)	2400 (1)	4000 (6)

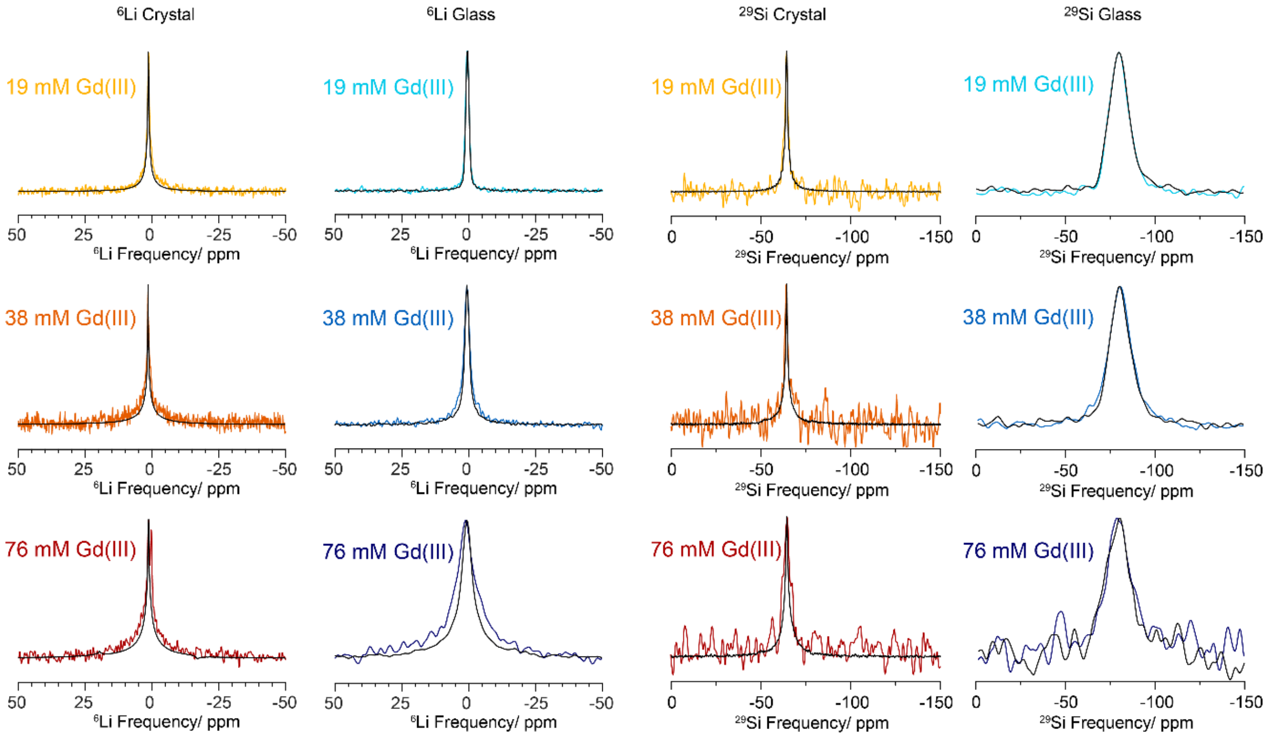

Figure S5 ^{6}Li and ^{29}Si NMR one-pulse and Hahn echo spectra obtained at approximately 100 K and a spinning speed of 9 kHz, with (black lines) and without (colored lines) microwave irradiation. Gd(III) content is given in the respective figures.

Table S6: DNP signal enhancements obtained from integrated areas.

Gd(III) concentration	Signal Enhancement $\varepsilon_{ON/OFF}$			
	Glass ^{6}Li	Glass ^{29}Si	Crystal ^{6}Li	Crystal ^{29}Si
19 mM	3.6	4.7	120.2	112.6
38 mM	4.8	5.1	90.0	91.4
76 mM	4.2	2.9	79.4	69.6

Variable MAS experiments

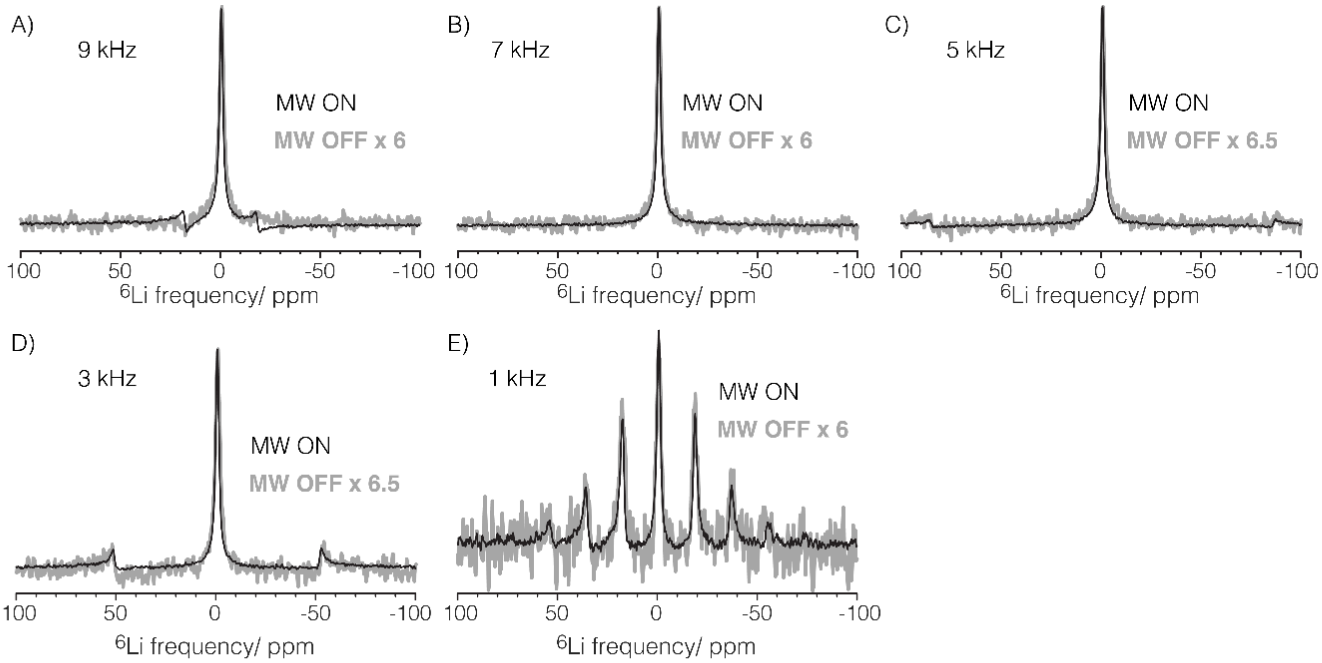

Figure S6 ^{6}Li one-pulse spectra of the $Li_2OCaO{\cdot}2SiO_2$ glass doped with 38 mM Gd(III). Spectra were acquired with a single scan after a recycle delay of 600 s at approximately 100 K and at variable spinning speeds, with (black lines) and without (grey lines). The slightly larger enhancements obtained in these set of measurements as compared to the one reported in Table S6 is likely a consequence of a recalibration of the gyrotron.

$T_{1,bu}$ Measurements

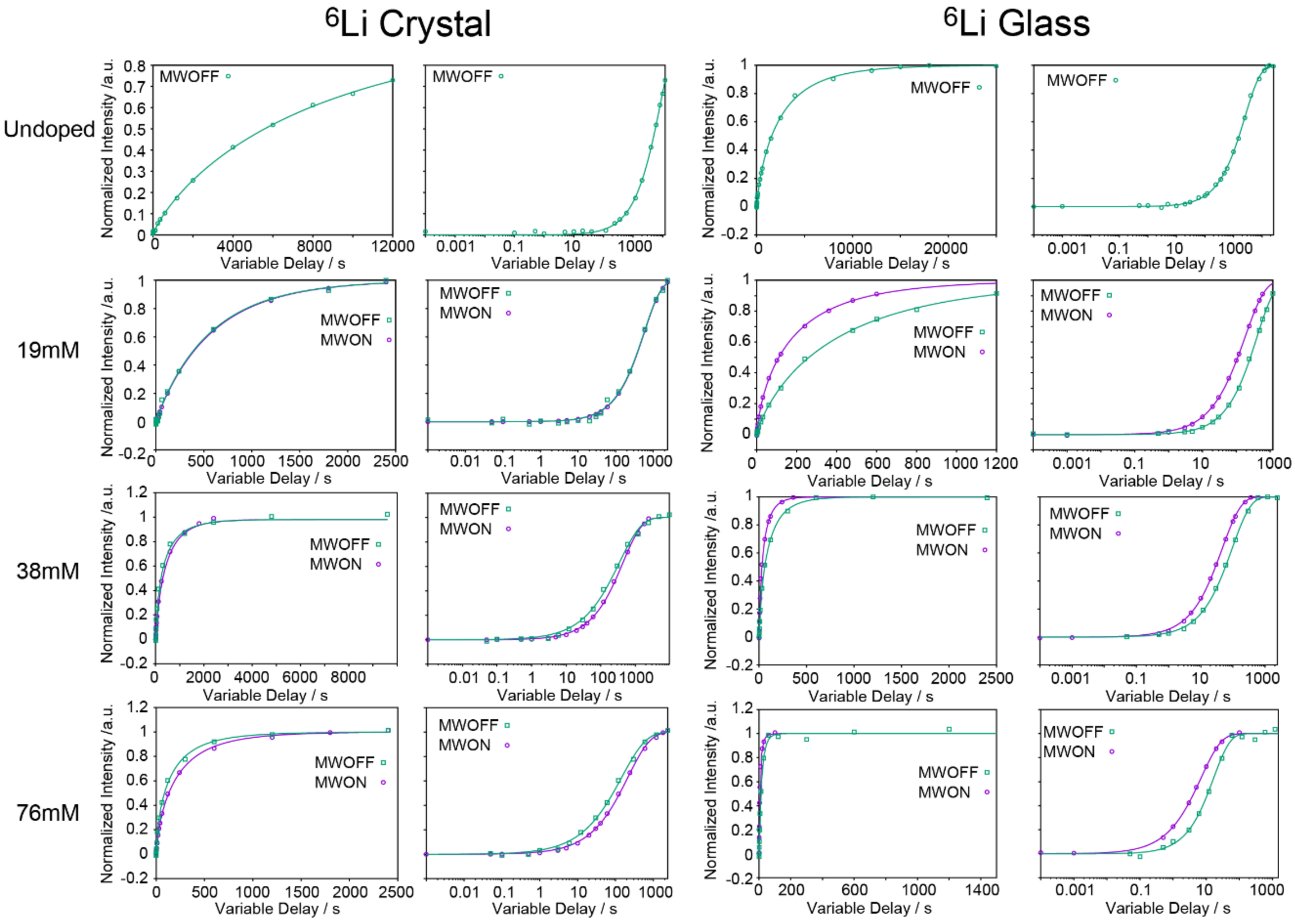

Figure S7 ^{6}Li magnetization buildup curves (linear and semi-log, left and right) in a saturation recovery experiment with (purple squares) and without (green circles) microwave irradiation, together with best fits (solid lines) obtained with a stretched exponential recovery according to equation (1) in the main document. Fit parameter are given in Table S7. Measurements performed at 100 K and spinning at 9 kHz.

Fitting of the relaxation and buildup curves required the use of a stretch exponential function with $\beta_1 < 1$. As an exception, the ^{6}Li longitudinal relaxation of the crystalline sample doped with 19 mM Gd(III) was best fit to an almost exponential recovery with $\beta_1 = 0.95 \pm 0.04$. A possible explanation for this could be that at this long relaxation time the spin diffusion among ^{6}Li nuclei is sufficiently efficient to ensure a large homogenization of the magnetization within the homonuclear spin bath.

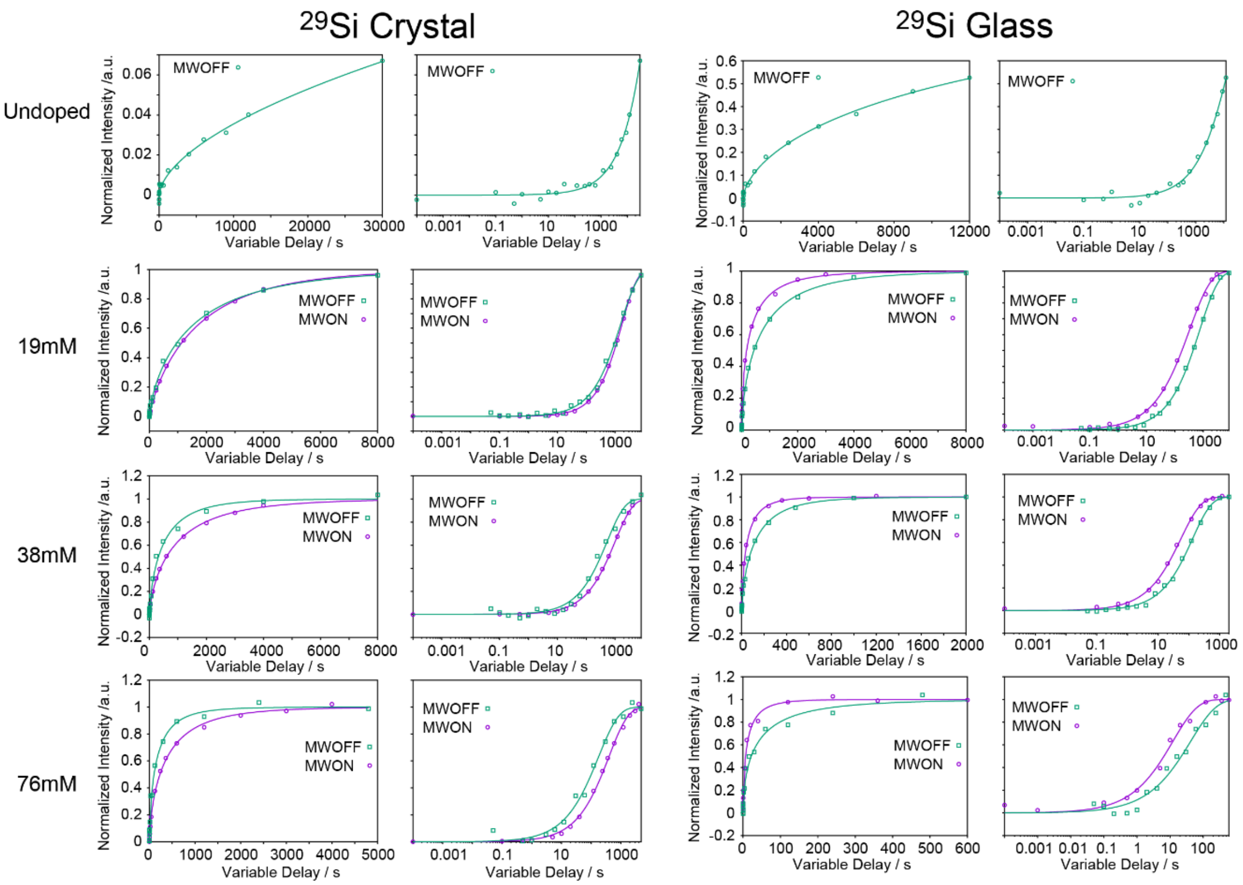

Figure S8 ^{29}Si magnetization buildup curves (linear and semi-log, left and right) in a saturation recovery experiment with (purple squares) and without (green circles) microwave irradiation, together with best fits (solid lines) obtained with a stretched exponential recovery according to equation (1) in the main document. Fit parameters are given in Table S7. Measurements performed at 100 K and spinning at 9 kHz.

Table S7: Best fit parameter of ^{6}Li and ^{29}Si longitudinal relaxation times obtained using equation (1) in the main document. Measurements performed at 100 K and MAS at 9 kHz.

**Gd(III) concentration**	**Glass ^{6}Li**			
	T_1 (s)	β_1	T_{bu} (s)	β_{bu}
undoped	2500±50	0.79±0.01	-	-
19 mM	410±20	0.81±0.02	181±6	0.73±0.01
38 mM	95±2	0.75±0.01	47±1	0.74±0.01
76 mM	18±1	0.86±0.06	7±0.2	0.70±0.01
	**Crystal ^{6}Li**			
	T_1	β_1	T_{bu}	β_{bu}
undoped	8700±1500	0.82±0.03	-	-
19 mM	570±40	0.95±0.04	580±10	0.96±0.01
38 mM	347±20	0.68±0.03	440±15	0.79±0.01
76 mM	144±10	0.58±0.02	215±7	0.73±0.01
	**Glass ^{29}Si**			
	T_1	β_1	T_{bu}	β_{bu}
19 mM	760±20	0.66±0.01	330±30	0.58±0.02
38 mM	131±7	0.71±0.02	55±3	0.66±0.02
76 mM	42±10	0.56±0.07	12±2	0.58±0.05
	**Crystal ^{29}Si**			
	T_1	β_1	T_{bu}	β_{bu}
19 mM	1590±150	0.73±0.03	1760±70	0.82±0.01
38 mM	530±70	0.71±0.05	1000±60	0.71±0.02
76 mM	180±30	0.64±0.05	400±20	0.67±0.02

T_2 Relaxation

We determined the coherence lifetimes by fitting the experimental data obtained from one pulse, Hahn echo and CPMG experiments to a stretched exponential:

$$M_{xy}(t) = M_{xy}(0)\exp\left(-(t\lambda)^{\beta_2}\right). \quad (1)$$

Where λ is the decay rate constant and β_2 the stretched exponent, as explained in the main document. We have assumed that λ^{-1} from the FID is a good measure of T_2 in two cases, and have used those values for comparison of T_2 relaxation times in Figure 4 of the main document: The CPMG of ^{29}Si in the 76 mM Gd(III) doped glass sample presented a large uncertainty, due to the fast decay leading to a small number of echoes measured. The T_2 obtained from the fit appears unrealistically long. As in this case the FID decay rate shows a markedly stretched behavior and is significantly shorter than in the lower doped samples, we assume that the decay rate from the FID gives a good estimate of T_2 in this sample. And second, the ^6Li lineshape of the 19 mM doped crystalline sample is clearly dominated by the presence of paramagnetic center (compare lineshape of undoped and doped samples in Figure 3 of the main document). Thus, also for this sample we estimated T_2 from the FID. This is further validated by the good agreement in λ^{-1} of ^6Li in 38 and 76 mM doped crystal, obtained from FID and Hahn echo experiment.

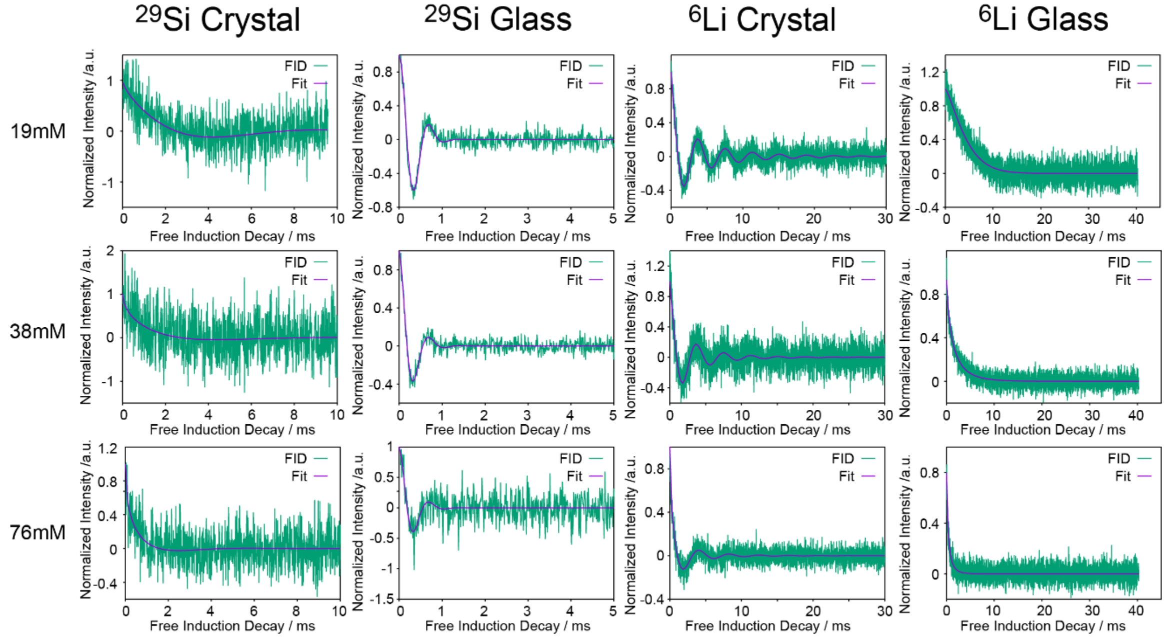

Figure S9 ^{29}Si and ^6Li FID obtained from a Hahn echo and a one-pulse experiment, respectively. Measurements performed at 100 K and spinning at 9 kHz. Also shown the best fits (purple lines) obtained with a stretched exponential decay according to equation (2) in the main document, multiplied by an oscillation, with the frequency obtained from the center of the signal after Fourier transform and maintained fixed in the fitting procedure. Fit parameters are given in Table S8.

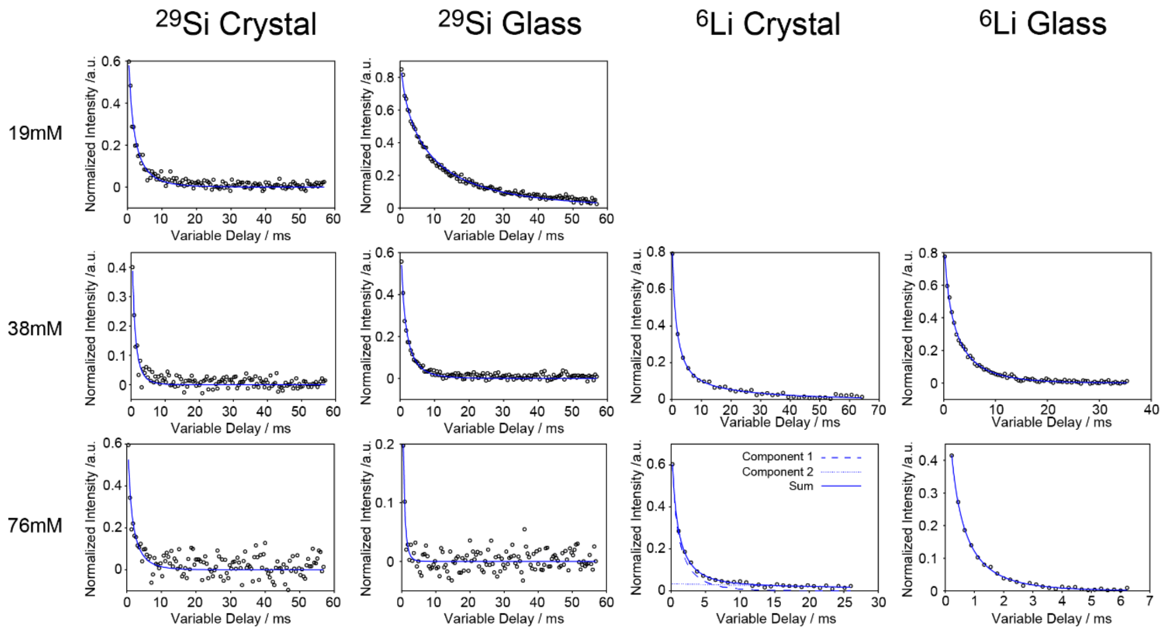

Figure S10 ^{29}Si and ^{6}Li CPMG and Hahn Echo decays, respectively. Measurements performed at 100 K and spinning at 9 kHz. Also shown the best fits (purple lines) obtained with a stretched exponential decay according to equation (2) in the main document. Fit parameters are given in Table S8. The ^{6}Li decay of the 76mM crystal sample required two exponential decays for a good fit (see discussion in main text).

Table S8: Best fit parameter of ^{6}Li and ^{29}Si coherence lifetimes λ^{-1} obtained using equation (2) in the main document, either from the free induction decay (λ^{-1}_{FID}) or the decay of the echo tops in a CPMG (for ^{29}Si) or a Hahn Echo (^{6}Li) experiment (λ^{-1}_{Echo}). The time $2\tau_1$ refers to the echo spacing in the CPMG or to the increment in the echo delay in the Hahn echo experiment. Values in bold were used in Figure 4 in the main document (see discussion in text). Measurements performed at 100 K and spinning at 9 kHz.

**Gd(III) concentration**	**Glass ^{6}Li**				
	$2\tau_1$ (ms)	λ^{-1}_{Echo} (ms)	$\beta_{2,Echo}$	λ^{-1}_{FID} (ms)	$\beta_{2,FID}$
19 mM		-	-	4.7±0.1	1.32±0.03
38 mM	0.44	**1.96±0.09**	0.64±0.02	1.29±0.05	0.71±0.02
76 mM	0.22	**0.28±0.03**	0.58±0.03	0.31±0.05	0.64±0.04
	**Crystal ^{6}Li**				
	$2\tau_1$ (ms)	λ^{-1}_{Echo} (ms)	$\beta_{2,Echo}$	λ^{-1}_{FID} (ms)	$\beta_{2,FID}$
19 mM		-	-	**1.7±0.1**	0.58±0.01
38 mM	1.78	**1.20±0.02**	0.6±0.1	1.6±0.1	0.67±0.03
76 mM	0.88	**0.68±0.01**	0.57±0.07	0.44±0.02	0.49±0.01
	**Glass ^{29}Si**				
	$2\tau_1$ (ms)	λ^{-1}_{Echo} (ms)	$\beta_{2,Echo}$	λ^{-1}_{FID} (ms)	$\beta_{2,FID}$
19 mM	0.44	**7.3±0.2**	0.6	0.52±0.02	1.8±0.1
38 mM	0.44	**1.00±0.04**	0.6	0.35±0.02	1.2±0.1
76 mM	0.44	**0.20±0.05**	0.6	0.36±0.06	1.3±0.4
	**Crystal ^{29}Si**				
	$2\tau_1$ (ms)	λ^{-1}_{Echo} (ms)	$\beta_{2,Echo}$	λ^{-1}_{FID} (ms)	$\beta_{2,FID}$
19 mM	0.44	**1.2±0.1**	0.6	1.6±0.4	0.7±0.1
38 mM	0.44	**0.49±0.06**	0.6	0.83±0.1	0.58±0.1
76 mM	0.44	0.9±0.2	0.6	**0.29±0.06**	0.58

The T_1/T_2 ratio

We estimated the rate of electron fluctuations from the ratio of T_1 over T_2 as described in a previous work.[1] This analysis requires that both nuclear relaxation times are governed by the same PRE and that the spatial part of the dipolar coupling is static. In addition, ideally spin diffusion should affect both relaxation mechanisms in an analogue way. This last point is likely not fully fulfilled for the ^{6}Li relaxation times in the crystalline sample as suggested from the large difference in the β values. As a consequence, the uncertainties of this approach will be larger than the ones obtained from simple error propagation of the fitting parameter of T_1 and T_2 , as given in the table. From the mean value across concentrations and taking into account both nuclei we estimate τ_{1e} as 0.8±0.2 and 2.0±0.5 μs in glass and crystal, respectively.

Table S9: Ratios of T_1/T_2 obtained from Table **S7** and **Table S8** (taken the values highlighted in bold) as well as τ_{1e} values calculated from the ratios and using equation 5 in the main text.

**Gd(III) concentration**	**Glass ^{6}Li**		**Crystal ^{6}Li**	
	T_1/T_2	τ_{1e} (μs)	T_1/T_2	τ_{1e} (μs)
19 mM	-	-	$3.3±0.3·10^5$	1.9±0.1
38 mM	$0.48±0.02·10^5$	0.7±0.02	$2.9±0.2·10^5$	1.8±0.1
76 mM	$0.62±0.08·10^5$	0.8±0.05	$2.1 ±0.2·10^5$	1.5±0.1
	**Glass ^{29}Si**		**Crystal ^{29}Si**	
	T_1/T_2	τ_{1e} (μs)	T_1/T_2	τ_{1e} (μs)
19 mM	$1.04±0.05·10^5$	0.79±0.02	$13±2·10^5$	2.8±0.2
38 mM	$1.31±0.08·10^5$	0.89±0.03	$11±2·10^5$	2.5±0.2
76 mM	$2.1±0.7·10^5$	1.1±0.2	$6±2·10^5$	1.9±0.3

DNP field sweeps

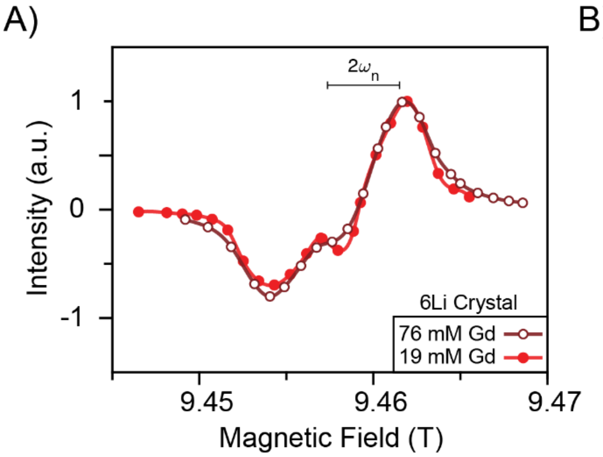

Figure S11 ^{6}Li DNP sweep profiles of crystal (A) and glass (B) samples doped with 76 mM Gd(III) (empty circles) shown in comparison with 19 mM Gd(III) doped samples (filled circles). Measurements performed at 100 K and spinning at 9 kHz.

3. EPR

EPR Spectra and Fits

The relative intensities of the different transitions in the EPR spectrum is strongly temperature dependent. Best fits were obtained using a temperature of 14 and 20 K for crystal and glass, respectively, in the simulations. This deviation from the set temperature of the experiment (10 K) might be a consequence of microwave heating, which affects the glass sample more than the crystalline. However, this large temperature raise is unexpected from the low power microwaves used, thus, an alternative explanation for the deviation in relative intensities could be related to differential transverse relaxation times, leading to a reduced echo signal intensity of the satellite transitions as compared to the central transition.

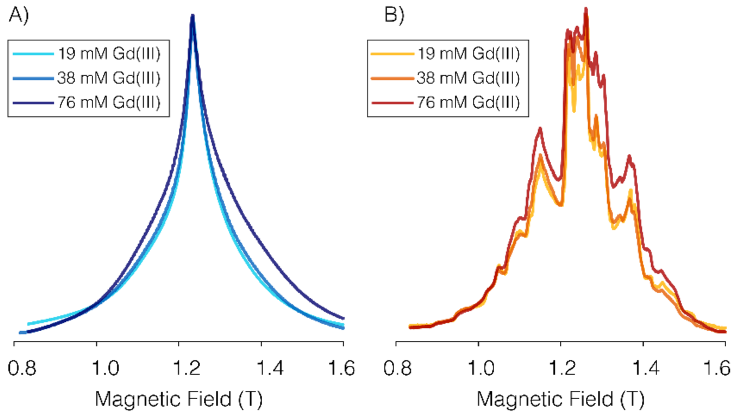

Figure S12 Experimental field sweep echo detected EPR spectra obtained at 10 K of glass and crystal for various Gd(III) doping levels at a microwave irradiation frequency of 35 GHz. At increased Gd(III) concentration, a broadening of the line is observed.

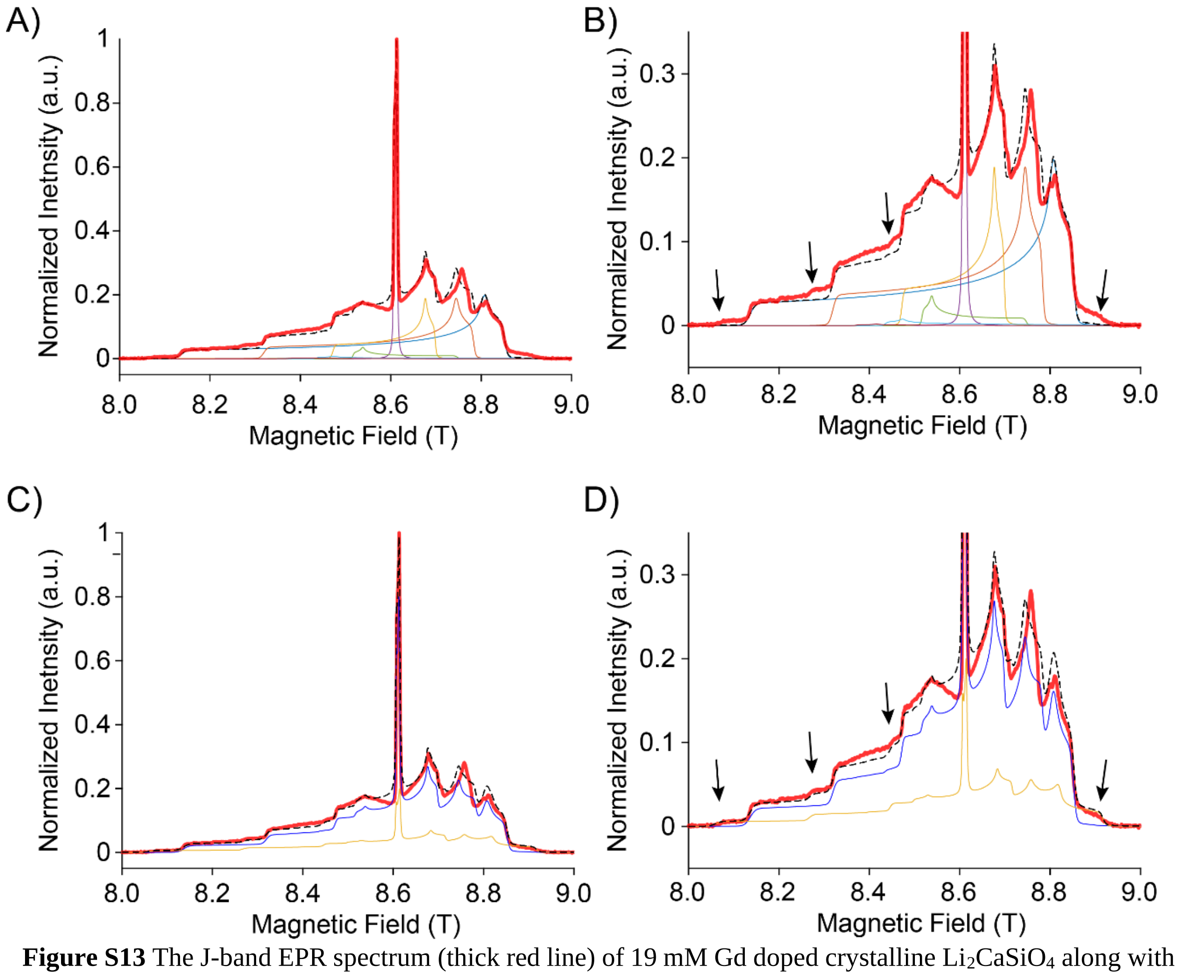

Figure S13 The J-band EPR spectrum (thick red line) of 19 mM Gd doped crystalline Li_2CaSiO_4 along with simulated spectra (dashed line), using one (A) and (B) or two (C) and (D) Gd(III) sites. (B) and (D) are respective vertical zooms of (A) and (C). In (A) and (B) the contribution of the various different transitions is shown as thin colored lines. From this figure the requirement of a second Gd(III) site is evident (regions improved by the second site are highlighted by the arrows). In (C) and (D) the contribution from both different sites from the fit shown in Figure 5 of the main document is shown in yellow and blue.

T_{1e} and T_{2e} Relaxation

Table S10: Best fit parameter of electron longitudinal and transverse relaxation times for glass and crystal samples doped with 19 mM Gd(III) and at a microwave irradiation frequency of 240 GHz, obtained using equations (1) and (2) in the main document.

**Temp (K)**	**Crystal**				**Glass**			
	T_{1e} (μs)	β_{1e}	T_{2e} (μs)	β_{2e}	T_{1e} (μs)	β_{1e}	T_{2e} (μs)	β_{2e}
4	50±3	0.51±0.01	2.99±0.01	0.83±0.01	-	-		
5	40±2	0.56±0.01	2.65±0.01	0.95±0.01	38.4±0.4	0.66±0.01	2.53±0.01	0.89±0.01
6	37.6±0.4	0.68±0.01	2.35±0.01	1.00±0.01	-	-	2.62±0.01	0.99±0.01
7	-	-	-	-	-	-	2.46±0.01	1.12±0.01
8	26.2±0.3	0.68±0.01	1.705±0.002	1.00±0.01	16.1±0.1	0.72±0.01	2.20±0.01	1.19±0.01
10	17.7±0.2	0.63±0.01	1.355±0.002	0.97±0.01	13.3±0.1	0.73±0.01	1.55±0.01	1.20±0.01
12	13.4±0.2	0.62±0.01	1.137±0.002	0.95±0.01	13.1±0.2	0.82±0.01	1.18±0.01	1.19±0.01
15	9.6±0.2	0.60±0.01	0.946±0.002	0.93±0.01	8.14±0.05	0.71±0.01	0.82±0.01	1.16±0.01
18	6.2±0.3	0.56±0.01	0.839±0.002	0.93±0.01	-	-	0.61±0.01	1.14±0.01
20	-	-	-	-	5.69±0.08	0.69±0.01		
21	5.4±0.1	0.56±0.01	0.763±0.001	0.93±0.01	--	-	0.478±0.001	1.12±0.01
25	4.5±0.1	0.57±0.01	0.698±0.001	0.93±0.01	4.3±0.1	0.71±0.01	0.364±0.001	1.10±0.01
30	4.19±0.06	0.61±0.01	0.621±0.001	0.94±0.01	2.8±0.1	0.68±0.02	0.278±0.001	1.08±0.01
35	3.88±0.08	0.64±0.01	0.566±0.001	0.94±0.01	-	-	0.261±0.001	1.08±0.01
40	3.5±0.1	0.66±0.02	0.517±0.003	0.96±0.01	1.6±0.1	0.67±0.03	0.214±0.001	1.05±0.01
45	-	-	-	-	-	-	0.185±0.001	1.05±0.01
50	2.7±0.1	0.67±0.02	0.415±0.003	0.98±0.01	1.41±0.1	0.68±0.05		
60	2.1±0.2	0.68±0.05	0.332±0.003	0.92±0.01	-	-		
80	-	-	0.240±0.003	0.95±0.01	-	-		

Table S11: Best fit parameter of electron longitudinal and transverse relaxation times for glass and crystal samples doped with 19 mM Gd(III) and at a microwave irradiation frequency of 120 GHz, obtained using equations (1) and (2) in the main document.

**Temp**	**Crystal**				**Glass**			
**(K)**	T_{1e} (μs)	β_{1e}	T_{2e} (μs)	β_{2e}	T_{1e} (μs)	β_{1e}	T_{2e} (μs)	β_{2e}
5	53.2±0.5	0.52±0.01	1.69±0.01	0.91±0.01	112±7	0.76±0.04	4.35±0.01	1.18±0.01
6					78±6	0.73±0.04	3.24±0.01	1.22±0.01
8	22.1±0.3	0.54±0.01	1.21±0.01	0.91±0.01	45.4±0.3	0.72±0.01	2.13±0.01	1.24±0.01
10	15.4±0.3	0.53±0.01	1.06±0.01	0.89±0.01	28.4±0.1	0.70±0.01	1.49±0.01	1.21±0.01
15	9.0±0.2	0.50±0.01	0.84±0.01	0.88±0.01	13.4±0.1	0.67±0.01	0.78±0.01	1.17±0.01
20	6.7±0.1	0.50±0.01	0.78±0.01	0.88±0.01				
30	5.3±0.1	0.52±0.01	0.69±0.01	0.87±0.01				
40	4.7±0.1	0.57±0.01	0.57±0.01	0.90±0.01				
60	2.9±0.1	0.65±0.02	0.40±0.01	1.00±0.01				
80	1.7±0.3	0.71±0.07						

Table S12: Best fit parameter of electron longitudinal and transverse relaxation times for glass and crystal at 10 K and a microwave irradiation frequency of 35 GHz, obtained using equations (1) and (2) in the main document.

**Gd(III) conc.**	**Crystal**				**Glass**			
	T_{1e} (μs)	β_{1e}	T_{2e} (ns)	β_{2e}	T_{1e} (μs)	β_{1e}	T_{2e} (ns)	β_{2e}
19 mM	15.8±0.1	0.56±0.01	546±4	0.98±0.01	19.7±0.1	0.68±0.01	573±2	1.20±0.01
38 mM	6.5±0.1	0.50±0.01	336±3	0.85±0.01	11.7±0.1	0.78±0.01	312±1	1.13±0.01
76 mM	9.8±0.1	0.50±0.01	282±3	0.90±0.01	1.8±0.1	0.73±0.01	88±1	1.01±0.01

References

(1) Jardón-Álvarez, D.; Malka, T.; van Tol, J.; Feldman, Y.; Carmieli, R.; Leskes, M. Monitoring Electron Spin Fluctuations with Paramagnetic Relaxation Enhancement. *J. Magn. Reson.* **2022**, *336*, 107143. https://doi.org/10.1016/j.jmr.2022.107143.
